# Automatic Extraction of Recurrent Patterns of High Dominant Frequency Mapping During Human Persistent Atrial Fibrillation

**DOI:** 10.3389/fphys.2021.649486

**Published:** 2021-03-12

**Authors:** Xin Li, Gavin S. Chu, Tiago P. Almeida, Frederique J. Vanheusden, João Salinet, Nawshin Dastagir, Amar R. Mistry, Zakariyya Vali, Bharat Sidhu, Peter J. Stafford, Fernando S. Schlindwein, G. André Ng

**Affiliations:** ^1^Department of Cardiovascular Science, University of Leicester, Leicester, United Kingdom; ^2^School of Engineering, University of Leicester, Leicester, United Kingdom; ^3^School of Science and Technology, Nottingham Trent University, Nottingham, United Kingdom; ^4^Biomedical Engineering, Centre for Engineering, Modelling and Applied Social Sciences (CECS), Federal University of ABC, Santo André, Brazil; ^5^Auckland Bioengineering Institute, University of Auckland, Auckland, New Zealand; ^6^National Institute for Health Research Leicester Cardiovascular Biomedical Research Centre, Glenfield Hospital, Leicester, United Kingdom

**Keywords:** atrial fibrillation, catheter ablation – atrial fibrillation, non-contact mapping, atrial electrograms, dominant frequency analyses, recurrent patterns, spatiotemporal patterns, pattern recognition

## Abstract

**Purpose:** Identifying targets for catheter ablation remains challenging in persistent atrial fibrillation (persAF). The dominant frequency (DF) of atrial electrograms during atrial fibrillation (AF) is believed to primarily reflect local activation. Highest DF (HDF) might be responsible for the initiation and perpetuation of persAF. However, the spatiotemporal behavior of DF remains not fully understood. Some DFs during persAF were shown to lack spatiotemporal stability, while others exhibit recurrent behavior. We sought to develop a tool to automatically detect recurrent DF patterns in persAF patients.

**Methods:** Non-contact mapping of the left atrium (LA) was performed in 10 patients undergoing persAF HDF ablation. 2,048 virtual electrograms (vEGMs, EnSite Array, Abbott Laboratories, USA) were collected for up to 5 min before and after ablation. Frequency spectrum was estimated using fast Fourier transform and DF was identified as the peak between 4 and 10 Hz and organization index (OI) was calculated. The HDF maps were identified per 4-s window and an automated pattern recognition algorithm was used to find recurring HDF spatial patterns. Dominant patterns (DPs) were defined as the HDF pattern with the highest recurrence.

**Results:** DPs were found in all patients. Patients in atrial flutter after ablation had a single DP over the recorded time period. The time interval (median [IQR]) of DP recurrence for the patients in AF after ablation (7 patients) decreased from 21.1 s [11.8 49.7 s] to 15.7 s [6.5 18.2 s]. The DF inside the DPs presented lower temporal standard deviation (0.18 ± 0.06 Hz vs. 0.29 ± 0.12 Hz, *p* < 0.05) and higher OI (0.35 ± 0.03 vs. 0.31 ± 0.04, *p* < 0.05). The atrial regions with the highest proportion of HDF region were the septum and the left upper pulmonary vein.

**Conclusion:** Multiple recurrent spatiotemporal HDF patterns exist during persAF. The proposed method can identify and quantify the spatiotemporal repetition of the HDFs, where the high recurrences of DP may suggest a more organized rhythm. DPs presented a more consistent DF and higher organization compared with non-DPs, suggesting that DF with higher OI might be more likely to recur. Recurring patterns offer a more comprehensive dynamic insight of persAF behavior, and ablation targeting such regions may be beneficial.

## Introduction

Atrial fibrillation (AF) is the most common cardiac arrhythmia in clinical practice, affecting 1–2% of the population worldwide (Nattel, 2002). AF increases the risk of stroke 5-fold and is associated with increased heart failure, mortality and higher healthcare utilization costs (Nattel, 2002). Although catheter ablation is an effective therapy for paroxysmal AF (pAF) (Haissaguerre et al., 1998; Fichtner et al., 2015), the identification of areas for successful ablation in patients with persistent AF (persAF) remains a challenge due to the existence of complex arrhythmogenic mechanisms (Jalife et al., 2002; Nattel, 2002, 2003).

**Figure 1:**
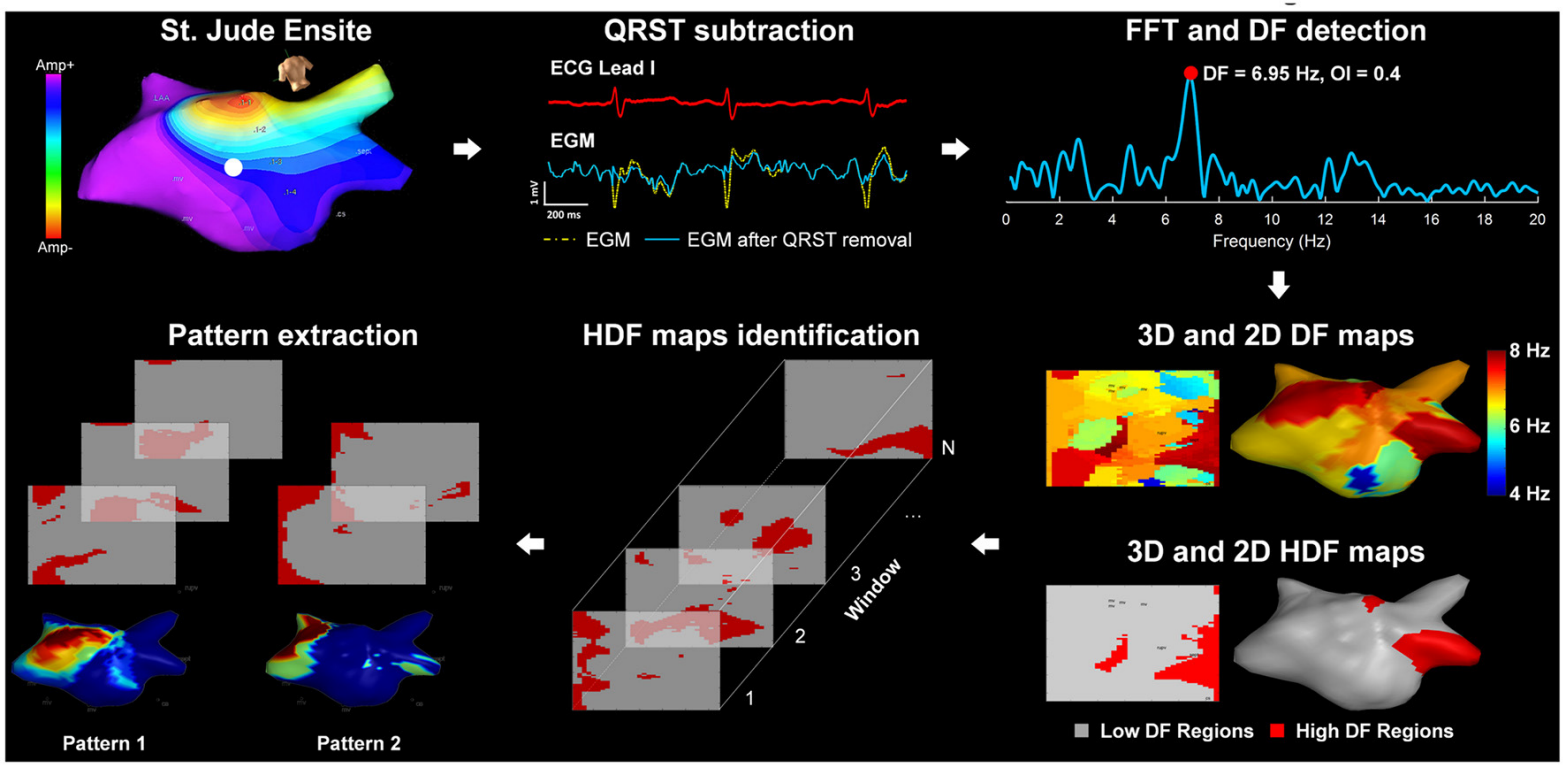
Diagram of the signal processing steps of the atrial electrograms: (1) St. Jude Ensite: left atrial Geometry isopotential map exported from Ensite Velocity System; (2) QRST subtraction: Electrograms using one ECG lead as reference; (3) FFT and DF detection: power spectrum of the current non-contact atrial signal and DF identification; (4) 3D and 2D DF/HDF maps: MATLAB reconstructed 3D Atrial geometry with color-coded DF/HDF and transformation to 2D uniform grid; (5) HDF maps identification: sequential 2D HDF maps used as input of the pattern extraction algorithm; (6) Pattern extraction: pattern extraction algorithm identifying recurrent spatial patterns.

Previous studies have shown that sustained AF induces structural and electrical remodeling in atrial tissue (Goette et al., 1996). These regions can potentially host focal ectopic activity and re-entry circuits, resulting in rapid local activations (Ashihara et al., 2012), which are important in triggering and perpetuating atrial arrhythmias. Atrial electrograms (EGMs) acquired from such atrial substrate regions have short cycle length. Dominant frequency (DF) has been introduced as a measure of the local activations from EGMs collected during AF (Figure 1) (Mansour et al., 2001). High DF (HDF) has been shown to represent atrial regions with rapid electrical activation rates, which might be related to remodeled atrial substrate and, therefore, could be targets for ablation (Sanders et al., 2005).

Clinical studies with DF-guided ablation reported suboptimal results. A small clinical study that first demonstrated combining pulmonary vein isolation (PVI) with ablation of maximum DF sites achieved a success rate of 56% in persAF patients (Atienza et al., 2009). Later, a prospective randomized clinical trial of 232 patients with paroxysmal and persistent AF reported no improvement in ablation outcomes with a DF-guided approach compared with PVI alone (Panikker et al., 2016). However, only sequential mapping approaches were used in those studies, with the implicit assumption of temporal stability of DF.

We have previously found that DF is spatiotemporally unstable when using high-density non-contact mapping (NCM) in the left atrium (LA) (Jarman et al., 2012). This finding questions the appropriateness of using sequential data collection in DF mapping. More interestingly, our recent work suggested that some HDFs might present recurrent behavior, in which HDF activity reappears in the same atrial region in different time instants (Salinet et al., 2014). Therefore, we hypothesize that atrial regions with recurrent HDF activity might provide further details of the underlying mechanisms that sustain AF. The main objective of this study is to develop an automated tool to investigate the recurrent behavior of HDF maps and its spatiotemporal repetition. Through detailed investigation of recurrent HDF maps, the underlying spatiotemporal periodicity of atrial activity could be unveiled and may offer a more comprehensive insight of dynamic behavior in underlying persAF and the impact of ablation.

## Methods

### Electrophysiological Study

The present USURP-AF (Understanding the electrophysiological substrate of persistent atrial fibrillation) study was approved by the local ethics committee at the University Hospitals of Leicester NHS Trust and local NHS research ethics committee. Ten patients undergoing catheter ablation for persAF for the first time were recruited. Details of the patients' baseline characteristics are presented in Table 1.

**Table 1:** Clinical characteristics of patients.

**Patient characteristics**			
*n* = 10 (male)			
Male (*n*)	10		
On amiodarone (*n*)	2		
	**Median**	**Min**	**Max**
Age (years)	57.8	36.1	76.4
Days in AF pre-procedure (days)	219	132	848
BMI	30.4	23.4	43.8
Previous DCCVs	2	1	5
Days since last DCCV	487	224	1237
Procedure time (mins)	390	309	475
Fluoroscopy (mins)	77.2	51.4	98.5
Ablation (mins)	44.1	26.5	109.2
Ablation area (mm^2^/% of LA)	1368 (7.5)	627 (3.3)	2668 (13.6)

Prior to the ablation, all drugs except amiodarone were stopped for at least 4 half-lives. During the procedure, bilateral femoral venous access was obtained under fluoroscopic guidance, and a quadripolar catheter and a deflectable decapolar catheter were placed at the His position and Coronary Sinus (CS), respectively. Trans-septal puncture was performed to gain access to the left atrium (LA). A non-contact multi-electrode array (MEA) catheter (EnSite Velocity, Abbott Laboratories, USA) and a conventional deflectable mapping catheter were deployed in the LA. Anticoagulant drugs were administered to maintain an activated clotting time >300 s. A high-resolution 3D geometry of the LA was created using EnSite Velocity electro-anatomical mapping system (Abbott Laboratories, Figure 1) and anatomical locations were annotated. Virtual EGMs (vEGMs) were recorded for up to 5 min. Baseline recording was collected for up to 5 min, from which a 30 s segment was exported and analyzed during the procedure to obtain the HDF sites in the LA using an in-house computer application (Li et al., 2017). High DF regions in the LA were identified as previously described (Salinet et al., 2014). The clusters of centers of HDF sites were targeted for ablation (Li et al., 2017) (Supplementary Figure 12). Following DF-guided ablation, a post-ablation recording was collected for up to 5 min, following which pulmonary vein isolation (PVI) was carried out with the circular, multipolar PVAC catheter (Medtronic Inc.). Four out of 10 patients had AF termination [3 atrial flutter (AFL, 1 sinus rhythm) by HDF ablation prior to PVI (Chubb et al., 2016)]. There were no adverse events in any of our 10 patients.

### Data Acquisition and Signal Processing

Intracardiac signals were collected using the non-contact MEA catheter as described above. The vEGMs (2,048 channels) were sampled at 2034.5 Hz and exported with the filter setting of 1–150 Hz. The data was analyzed offline using MATLAB R2013a (Mathworks, USA). The vEGMs were resampled to 512 Hz using cubic spline interpolation method to reduce processing time while maintaining a relatively high sampling rate for the frequency analysis. As illustrated in Figure 1, ventricular far-field activity was removed from the recorded vEGMs using a QRST subtraction technique previously described (Salinet et al., 2013). The vEGMs were then divided into 4 s window segments with a 50% overlap. For each segment, spectral analysis was performed using fast Fourier transform (FFT). A Hamming window was applied to the atrial vEGMs to reduce leakage. Zero padding was used to improve the DF identification with a resulting frequency step of 0.05 Hz. DF was defined as the peak in the power spectrum within the physiological range of 4–10 Hz (Salinet et al., 2014).

### HDF Pattern Extraction

HDFs were defined as atrial nodes with DF values equal to or greater than the top 10th percentile DF values across the LA surface for each time window. Binary HDF maps were generated for all windows, where HDF regions were marked as “ones” and low DF (LDF) regions were marked as “zeros” (Figure 1). The vEGMs of 2,048 virtual electrodes were mapped onto a 64 × 32 2D rectangular grid, as previously described (Li et al., 2020a). The 2D binary HDF maps (graphic pattern) were used as the input of the pattern extraction algorithm based on 2D correlation (Li et al., 2015), to find the reappearing HDF maps in time and cluster the maps into patterns.

A 2D Pearson's correlation coefficient (CORR) (Equation 1) was used as a measurement of similarity between the HDF maps at different time windows. Here, *A, B* represent the 2D HDF maps; $\bar{A},\bar{B}$ their average values; and *i* and *j* are the row and column of the images.

(1) $$\begin{array}{l} CORR=\frac{\sum \sum \left(A_{ij}-\bar{A}\right)\left(B_{ij}-\bar{B}\right)}{\sqrt{\left({\sum \sum \left(A_{ij}-\bar{A}\right)}^{2}\right)\left({\sum \sum \left(B_{ij}-\bar{B}\right)}^{2}\right)}} \end{array}$$

**Figure 2:**
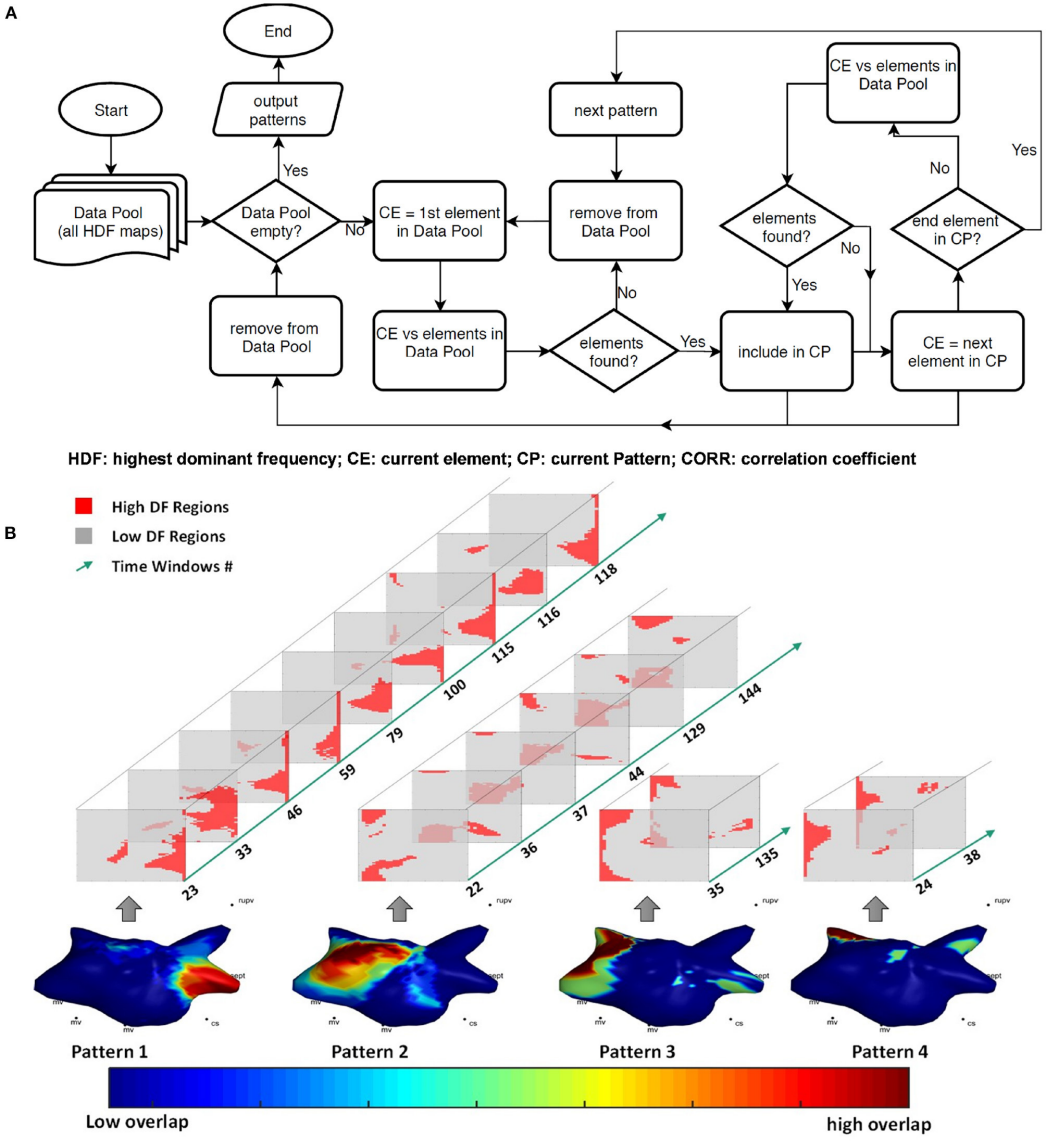
**(A)** The flowchart of the pattern extraction algorithm; **(B)** all recurrent patterns of patient 6 with the 2D HDF maps involved in each pattern and the corresponding time windows numbers, color-coded 3D geometry showing the overlap of all the pattern windows.

Figure 2A contains the flowchart of HDF pattern extraction algorithm, in which all the generated 2D HDF maps are referred to as the “data pool” and a single HDF map is referred to as an “element.” Briefly, the key steps of the algorithm can be explained as:

- Step 1: Compute the CORR of first element of the data pool with the rest, set the elements with CORR greater than a threshold and the current element (CE) defined as current pattern (CP).
- Step 2: Calculate the CORR of the other elements (if there are any) in CP with the elements in the data pool and add the elements greater than the threshold to CP. Remove the CP maps from the data pool, repeat this until there are no elements joining the CP at the last element.
- Step 3: Consider as a pattern if more than one element is found and move on to the next pattern.
- Step 4: Repeat steps 1, 2, and 3 until there are no more elements in the data pool. Sort the patterns by number of elements.

The CORR threshold was set as 0.6 for the analysis. Effects of the CORR threshold of the pattern extraction algorithm are discussed in the Supplementary Material. Please note the algorithm generates consistent patterns with arbitrary choice of first element of the data pool, as pattern elements will finally converge when no elements outside the pattern can generate CORR greater than threshold with any element in the current pattern.

### Temporal Pattern Analysis

The pattern extraction algorithm was applied to the HDF maps of the 10 patients before and after DF-guided ablation. Figure 2B illustrates one example of all the patterns for one patient. In order to investigate the recurrence of events, the dominant pattern (DP) was defined as the HDF pattern with the highest recurrence (Pattern 1).

### Feature Analysis of the Dominant Pattern

The mean and standard deviation of the DF, and the organization index (OI) of the HDF regions of each map were calculated. OI is defined as the area under the curve of DF peak divided by the area under the curve of the entire power spectrum (Everett et al., 2001; Jarman et al., 2014). The average and standard deviation of DF and OI of the DP windows and the non-DP windows were compared.

To study the behavior of secondary DPs, the mean and standard deviation of the DF, and the organization index (OI) of the HDF regions of each map were calculated. The DF and OI in the HDF regions of the secondary DPs (i.e., 2nd dominant, and 3rd dominant patterns) were compared. The average and standard deviation of DF and OI of the DP windows, secondary DPs and the non-DP windows were compared.

### Regional Analysis

The LA geometries of the 10 patients were manually segmented by an experienced clinician into 12 regions – Mitral valve (MV); Left upper pulmonary vein (LUPV); Left lower PV (LLPV); Right upper PV (RUPV); Right lower PV (RLPV); Roof; Posterior (Pos); Anterior (ant); LA appendage (LAA); MV isthmus (MVI); Septum (sept); Floor. The segmentation was manually performed on the EnSite Velocity System by creating virtual 3D surface lesion points to form closed loop boundary points for each anatomical region. The numbering of 3D locations was recorded and saved in Microsoft Excel format. An automated MATLAB software was developed to read the data and the corresponding 3D coordinates (lesion files) of the boundary lesion points. For each set of the boundary lesion points, a closed 3D polygonal mesh was created using Delaunay triangulation. Vectors were created connecting the center point of the LA mesh and surface nodes locations, and regional labels were estimated according to the intersection between the vectors and any of the triangular faces of the 3D polygon mesh. Thus, surface nodes of the triangular mesh within boundary points were detected for all regions, and all vEGMs were assigned to anatomical labels. The DP HDF occurrence at all anatomic regions were counted.

### Statistical Analysis

Wilcoxon matched-pairs signed rank tests were performed to compare the mean time intervals before and after ablation. The average and standard deviation of DF and OI of the DP windows and the non-DP windows were also compared using Wilcoxon matched-pairs signed rank tests. DF and OI of the DP, secondary DPs and non-pattern windows were compared with unpaired ordinary one-way ANOVA, and each two classes were compared with unpaired *t*-tests. The average and standard deviation of DF and OI of DP, secondary DPs and non-pattern windows were compared with paired one-way ANOVA, and each two classes were compared with paired *t*-tests. One-way ANOVA was used to compare the regions hosting DP. A *P*-value below 0.05 was considered significant in all statistical tests.

## Results

A total of 2,793 DF maps were analyzed, in a total of 2983.5 s before and 2664.5 s after DF-guided ablation. Recurrent patterns were found on all 10 patients before and after ablation using the proposed algorithm.

### Recurrence of HDF Patterns

**Figure 3:**
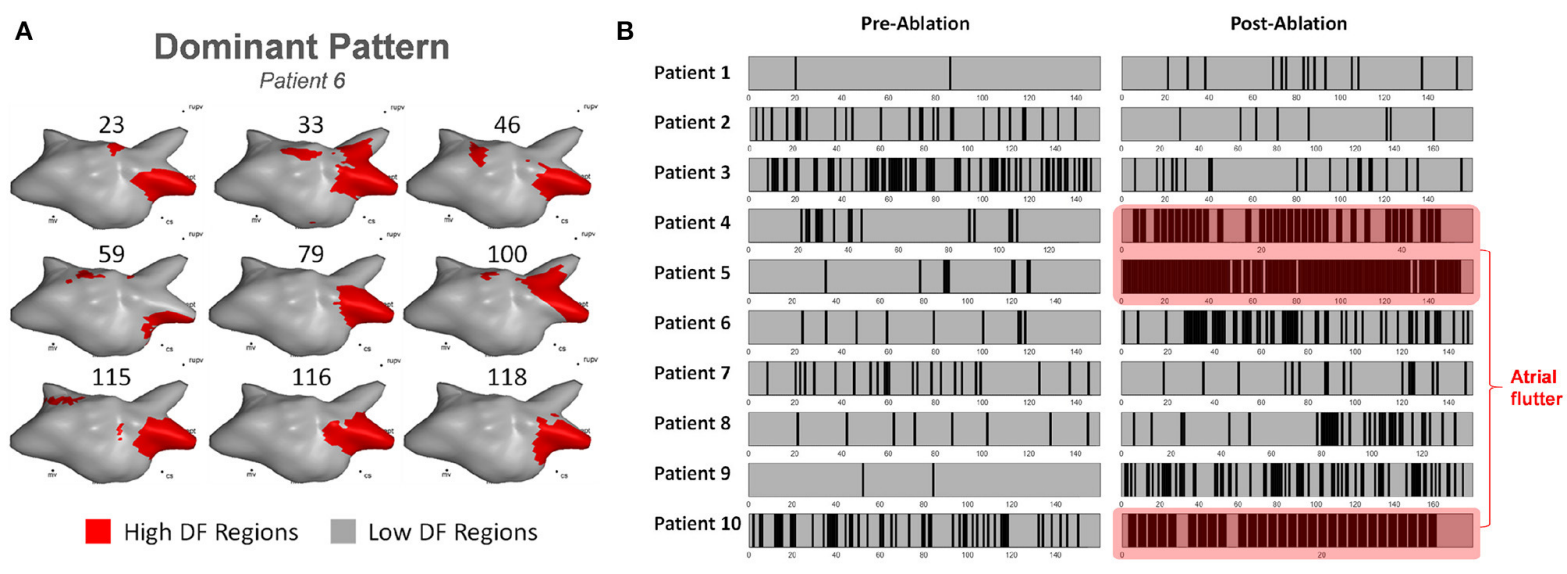
**(A)** Example of 3D HDF maps of the DP in patient 6; **(B)** The time occurrences of the DP of each patient pre-/post-ablation (black: DP windows; gray: non-DP windows). Patients 4, 5, and 10 were in atrial flutter post ablation demonstrating a single DP.

The time windows in which the DPs occurred were annotated before and after ablation for all the patients in order to investigate their periodic behaviors. An example of the DP recurrences and their atrial locations for one of the patients is illustrated in Figure 3A. The temporal behavior of the DPs for each patient is shown in Figure 3B. The time instants in which the DPs occurred are marked in black. Both the percentage of duration of the DP windows and the mean intervals between the time windows hosting DPs have been measured in order to assess the incidence of DP recurrences (Table 2).

**Table 2:** The duration of the dominant pattern windows and the mean interval among subsequent occurrences of patterns before and after ablation for all patients.

**Patient**	**% DP duration**		**Mean interval (s)**	
	**Pre**	**Post**	**Pre**	**Post**
1	1.4	9	132.00	20.31
2	17.4	4.9	11.23	37.14
3	43.2	12.7	4.38	15.68
4	11.5	68.1(AFL)	12.29	2.77
5	5.7	94.8 (AFL)	23.25	2.11
6	6.9	40.5	21.11	4.98
7	15.8	11.5	12.45	16.13
8	5.4	24.6	35.43	8.00
9	1.3	7.2	64.00	4.81
10	32.5	93.6 (AFL)	6.04	2.14
Median [IQR] All patients	9.2 [5.4 ~ 17]	18.6 [9.6 ~ 61.2]	16.8 [11.5 ~ 32.4]	6.5 [3.3 ~ 16.0]
Median[IQR] AF only	6.9 [3.4 ~ 16.6]	11.5 [8.1 ~ 18.7]	21.1 [11.8 ~ 49.7]	15.7 [6.5 ~ 18.2]

*AFL, atrial flutter; IQR, Interquartile Range*.

HDF-guided ablation therapy prior PVI increased the DPs occurrence in 7 out of 10 patients, suggesting increased AF organization following ablation, as illustrated in Figure 3B. In general, the time interval (median [IQR]) of DP recurrence for all patients decreased from 16.8 s [11.5–32.4 s] to 6.5 s [3.3–16.0 s] after ablation (*p* = 0.13). Patients 4, 5, and 10 converted to atrial flutter following ablation. The post-ablation DP accounted for 68.1% for patient 4, 94.8% for patient 5 and 93.6% for patient 10 of the total recorded windows, which corroborates the robustness of the method in capturing underlying recurrent DF patterns (Table 2). The time interval of DP recurrence for the patients remaining in AF (excluding patients 4, 5, and 10) after ablation decreased from 21.1 s [11.8–49.7 s] to 15.7 s [6.5–18.2 s] (*p* = 0.29).

Please note, as mentioned in section HDF Pattern Extraction, the CORR threshold was set as 0.6 for the analysis to allow a sufficient number of recurrences for all patients (see Supplementary Figure 11). This choice may have some effects on the above results of recurrences.

### DP Features

**Figure 4:**
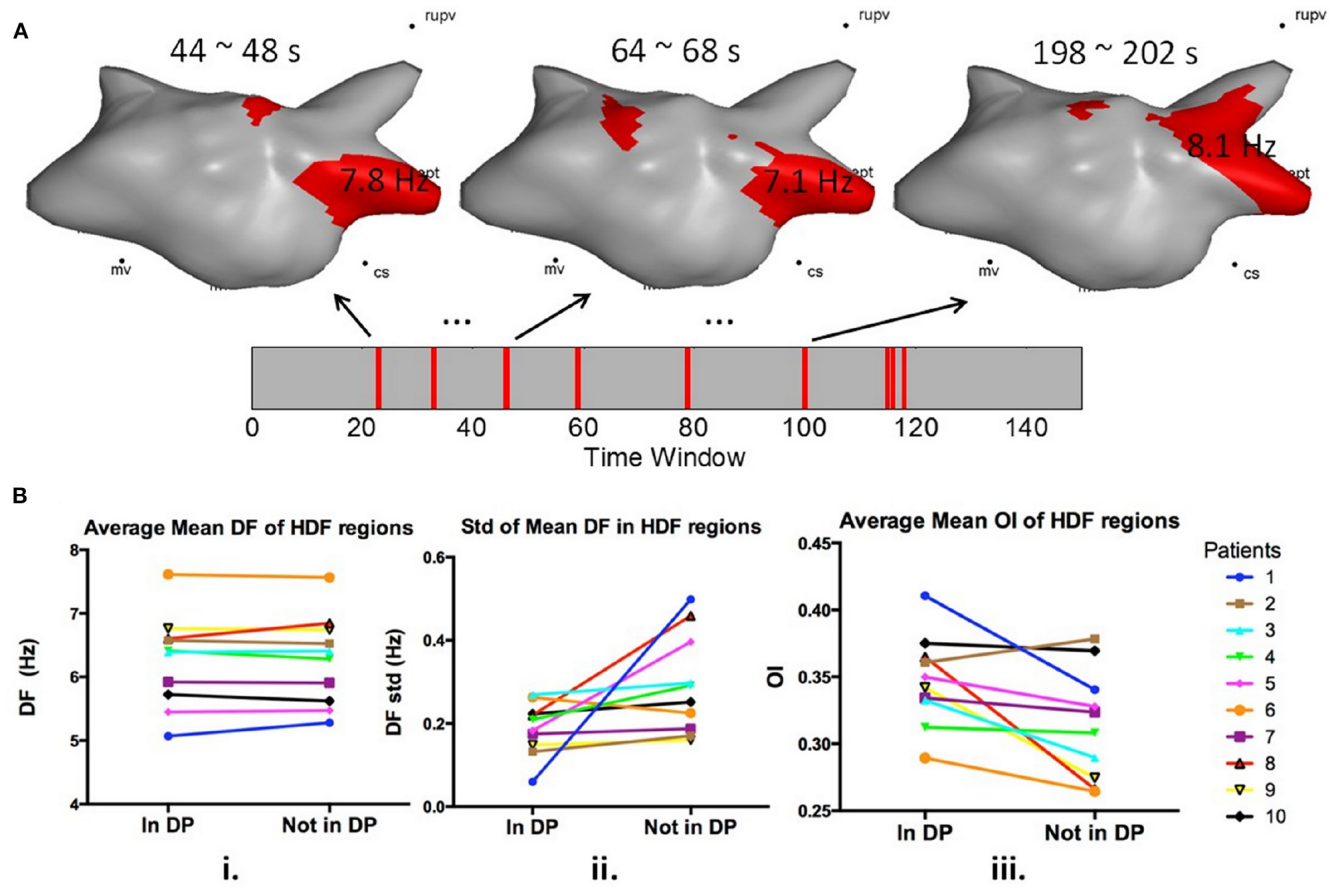
**(A)** 3D left atrial HDF maps in dominant pattern windows; **(B) i**. Average mean DF of HDF regions in and outside DP windows before ablation; **ii**. Standard deviation of mean DF of HDF regions in and outside DP windows; **iii**. Average mean OI of HDF regions in and outside DP windows before ablation.

The DF and OI in the HDF regions for all the maps were calculated. Figure 4A illustrates the recurring HDF maps and the time window occurrences of the DP. The average frequency in HDF regions inside the DPs was 6.25 ± 0.73 Hz, and 6.26 ± 0.70 Hz in non-DPs (*p* = 0.92; Figure 4Bi). However, the standard deviation of mean DF in HDF regions in the DPs was significant lower when compared with that outside the DPs (0.18 ± 0.06 vs. 0.29 ± 0.12 Hz, *p* < 0.05; Figure 4Bii). Additionally, the OI was significantly higher in the DP regions compared to non-DP regions (0.35 ± 0.03 vs. 0.31 ± 0.04, *p* < 0.05; Figure 4Biii).

### Regional Difference of DP Occurrence

**Figure 5:**
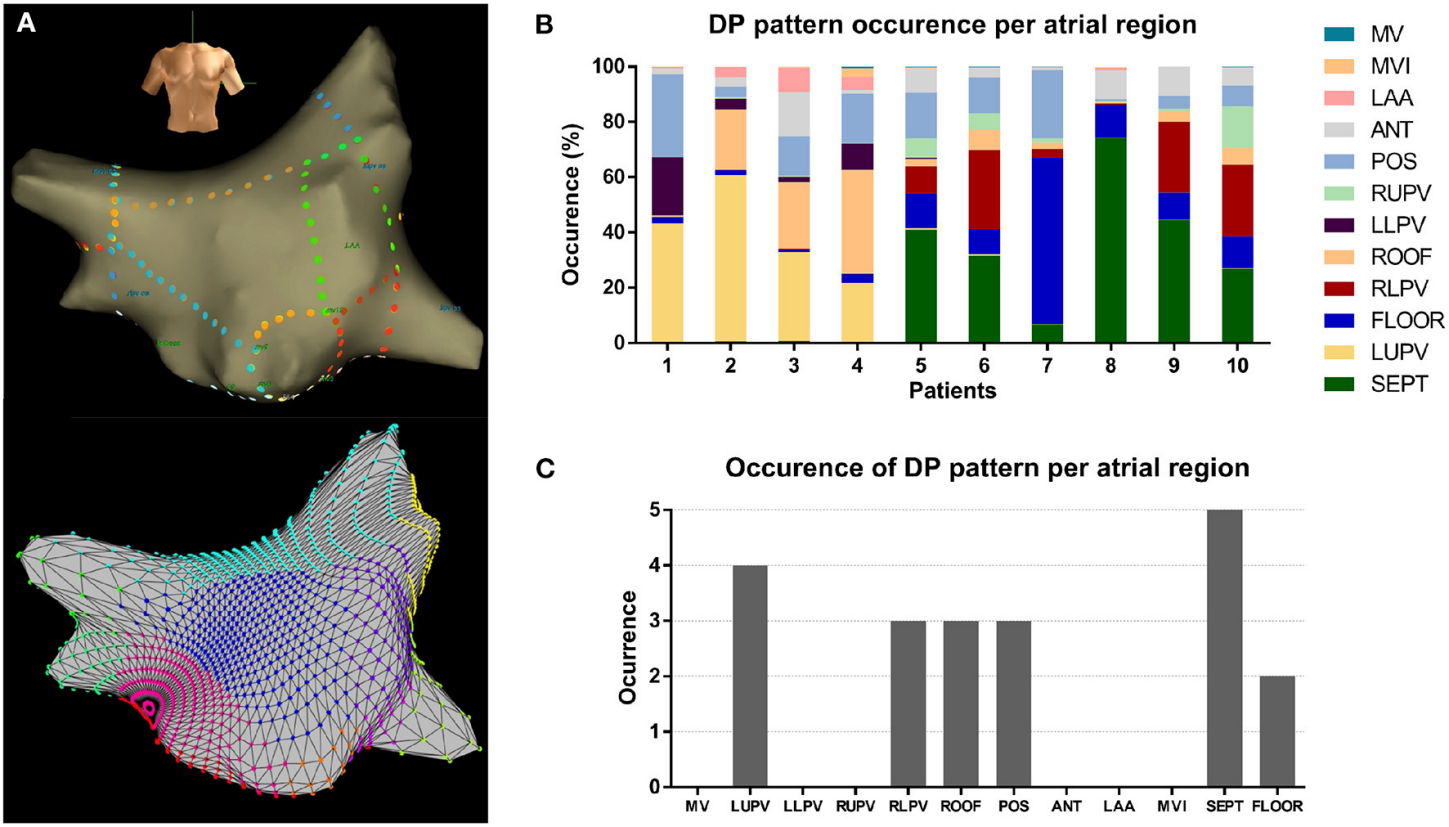
**(A)** Example of the atrial segmentation and detection (top: Ensite; bottom: MATLAB reconstruction); **(B)** Percentage of DP pattern occurrences of all patients at different atrial regions; **(C)** Count of occurrences of DPs of all patients at different atrial regions.

As illustrated in Figure 5A, the anatomic regions were segmented and HDF visits from DPs for each region were calculated. The proportion of HDF regions in DP on each anatomic region are summarized in Figure 5B. The distributions varied among regions (*p* < 0.01). The regions hosting DPs most often were the septum and the LUPV (Figure 5C).

### Secondary DP Features

**Figure 6:**
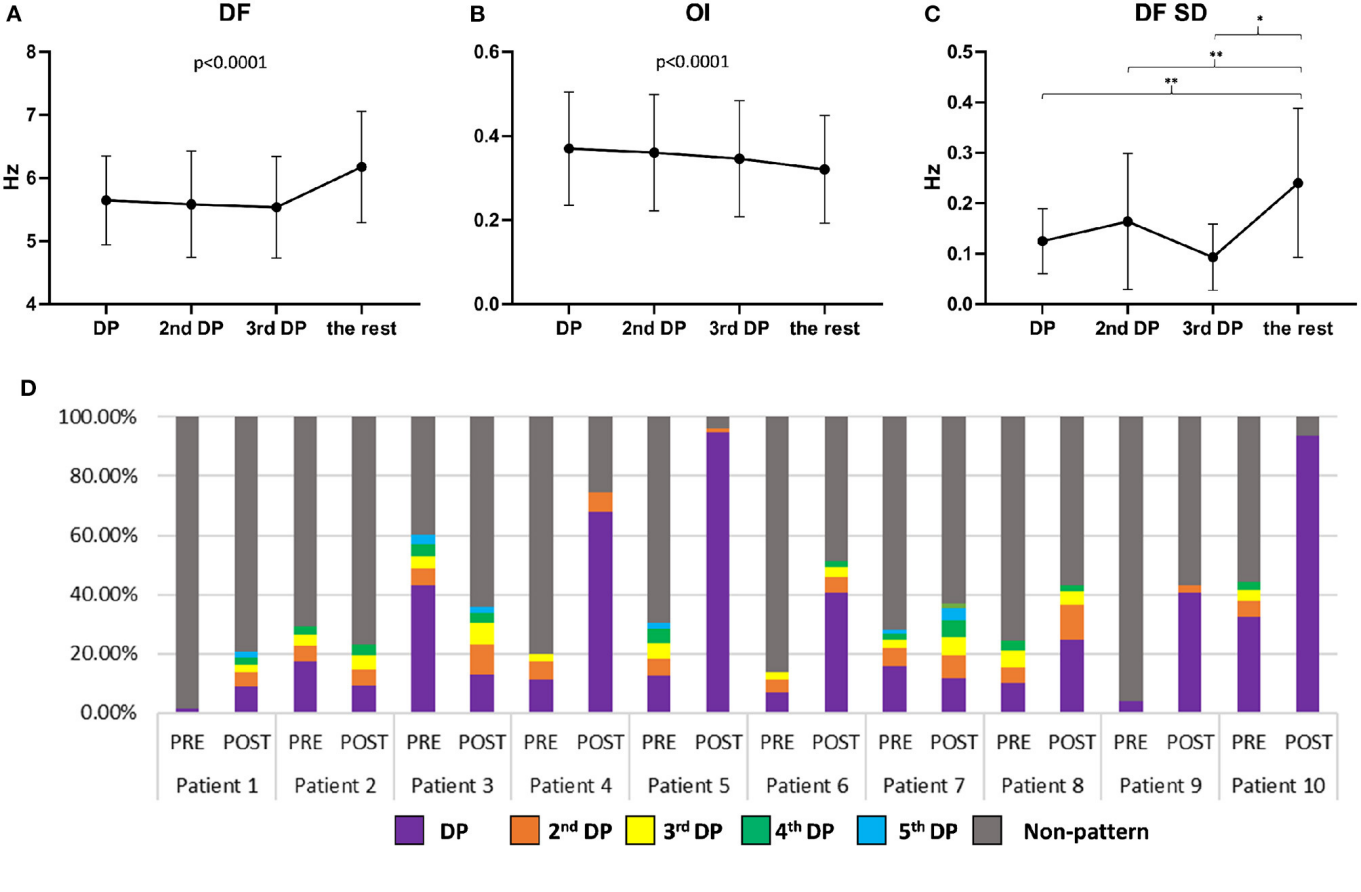
**(A)** DF values of HDF regions in 1st DP, 2nd DP, 3rd DP, and non-DP for all patients (mean and standard deviation); **(B)** OI values of HDF regions in 1st DP, 2nd DP, 3rd DP, and non-DP for all patients (mean and standard deviation); **(C)** Standard deviation of mean DF of HDF regions in 1st DP, 2nd DP, 3rd DP, and non-DP for all patients (mean and standard deviation). **(D)** The percentage of DPs and Non-DP occurrences for all patients before and after ablation.

As illustrated in Figure 6A, the DFs inside the HDF regions of the DP, 2nd DP, 3rd DP windows and non-DP were significantly different (5.65 ± 0.70 vs. 5.59 ± 0.85 vs. 5.54 ± 0.81 vs. 6.18 ± 0.88 Hz, *p* < 0.0001). DFs were shown to reduce with the decreased hierarchy of patterns (i.e., from 1st DP to 3rd DP, *p* < 0.0001), but were all significantly smaller than the non-DP windows (*p* < 0.0001). Figure 6B demonstrates that OIs inside the HDF regions were significantly different (DP: 0.37 ± 0.13; 2nd DP: 0.36 ± 0.14; 3rd DP: 0.35 ± 0.14; and non-DP: 0.32 ± 0.13, *p* < 0.0001). OIs directly decreased according to the decreasing order of dominance of the patterns (i.e., from 1st DP to 3rd DP, and then to non-DP windows, *p* < 0.0001). In Figure 6C, the standard deviation of mean DF in HDF regions across pattern windows were 0.125 ± 0.065 Hz for DP, 0.164 ± 0.135 Hz for 2nd DP; and 0.093 ± 0.066 Hz for 3rd DP (*p* = 0.1774 for DP vs. 2nd DP; *p* = 0.3558 for DP vs. 3rd DP; *p* = 0.2135 for 2nd DP vs. 3rd DP), but they were significantly lower when compared with the non-DP individually (0.240 ± 0.148 Hz; *p* = 0.0033 for DP vs. non-DP; *p* = 0.0016 for 2nd DP vs. non-DP; *p* = 0.0274 for 3rd DP vs. non-DP). As illustrated in Figure 6D, multiple patterns existed in all patients and a higher portion of DPs and secondary DPs were observed after ablation in most of the patients. Details of all the recurrent patterns from all patients can be found in the Supplementary Material.

## Discussion

In this study, we developed an automated tool that characterizes the recurrent behavior of HDF maps and its spatiotemporal repetition. We are the first to show that different patterns of DF consistently reappear in the LA during human persAF, providing valuable insights regarding the underlying spatiotemporal behavior and complexity of this arrhythmia. Although persAF is characterized by turbulent activations, the recurrence of DF patterns could indicate AF drivers which may have been anchored in the substrate. With detailed investigation of recurrent HDF maps, the underlying spatiotemporal periodicity of atrial activity could be unveiled and may offer a more comprehensive insight of the dynamic behavior of persAF. We have also shown that there exist recurrent spatial patterns at different levels of periodicity and present summaries of the types of spatial patterns that may be more likely to recur. Detailed investigations of these recurrent spatial patterns could further help to define the potential targets for ablation.

### HDF Pattern Recurrences

Previous studies have shown that the DFs of individual vEGMs are temporally unstable when mapped with NCM, where a recurrent behavior in HDF reappearance was noticed in the LA, sometimes within 10 s (Salinet et al., 2014). In the present study, multiple recurrent activities were found for most of the patients using the proposed pattern extraction technique on NCM signals. This finding reinforces the idea that persAF is not entirely random, and that instead, various degrees of spatiotemporal organization may co-exist (Gerstenfeld et al., 1992; Botteron and Smith, 1996). Despite the evident complexity of AF sustained by the meandering of multiple wavelets (Wijffels et al., 1995), wave collision (Allessie et al., 1996), or breakthrough activations (Gray et al., 1996), some atrial regions present remarkable recurrent activation with a certain degree of organization. These activities could therefore be explained by ectopic activities (Haissaguerre et al., 1998; Chen et al., 1999) and local re-entries (Moe et al., 1964). Additionally, one single DP was found in patients whose AF converted to atrial flutter following HDF-guided ablation. This was expected since atrial flutter is an organized arrhythmia compared to AF (Winter and Crijns, 2000). Atrial flutter is usually sustained by one macro re-entrant circuit and, therefore, the organized spatiotemporal behavior – i.e., faster and with more reappearances – of the DPs correlates with the higher level of regularity of atrial flutter. This finding could also support and help validate the potential use of the proposed method in describing spatiotemporal regularization from AF to more organized tachyarrhythmia. In addition, an overall increase of DP recurrence was found after ablation, suggesting catheter ablation in this cohort of patients may have increased the spatiotemporal regularization and transformed a complex persAF rhythm to a less complex and more organized tachyarrhythmia. Nevertheless, patients 4 and 5 had lowest DPs occurrences prior to ablation responded well to ablation, which may suggest that a less organized rhythm could still be converted with appropriate ablation.

### DP Features – What Frequency Features Are More Likely to Recur?

To investigate whether specific EGM features could predict HDF recurrence, binary HDF maps were set as input of the algorithm, so that the system was blind to the DF values of each HDF map. In other words, only the location and shape of the HDF maps were taken in consideration with regard to studying the recurrence behavior. Although the reappearing HDF maps were not dependent on the DF value that defined the HDF region, lower standard deviations of the mean DF within the DPs were found, which suggests that higher temporal consistency of the detected pattern over the non-pattern maps (i.e., when a map is recurrent in time, there is a high chance that the DF would be more similar than other non-recurring maps). OI has been proposed as a method to measure the “dominance” of the DF in atrial sites (Everett et al., 2001; Jarman et al., 2014). Our findings show the OI of the recurrent HDF maps to be higher in DP than in non-DP regions. HDF maps with higher OI being more likely to reappear further supports the hypothesis of the existence of underlying periodic atrial activity during AF. This may suggest that more organized periodic atrial activity may be more likely to drive the rhythm and might have a higher chance of showing a recurring spatial pattern.

In the analysis of secondary patterns, we found that the recurrent patterns did not necessarily have the highest frequency across all time windows. This could suggest that the higher non-recurrent frequency could be a passive phenomenon (wave collisions) (Allessie et al., 1996; Gray et al., 1996) and an unwanted harmonic frequency (multiples of the fundamental frequency) (Traykov et al., 2012). This was also supported by the results that spectral organization decreased from the most recurring pattern to less recurrent and dropped to a minimum at non-recurring patterns (Figure 6B). There was also a decrease in DFs from the most recurrent pattern to the least recurrent pattern, suggesting that patterns with high organization and relative high frequency could be more likely to recur in time with similar location and graphic pattern.

### Regional Pattern Distribution

The distribution of the recurrent DP areas varied among atrial regions for all patients. Our results suggest that the LUPVs and the septum are more likely to host DPs. Considering that PVs have been shown important in the initiation and maintenance of AF, and that usually maximum DF sites can be found near the PVs (Oral et al., 2002; Sanders et al., 2005; Atienza et al., 2009), it could be expected that the PVs might also be preferred sites to host DPs. Additionally, our findings suggest that the septum is one of the regions prone to host recurrent behaviors, which is in agreement with previous studies that have shown the septum as one of the critical sites for AF termination during ablation (Schmitt et al., 2007; Calo et al., 2008; Porter et al., 2008; Takahashi et al., 2008; Park et al., 2009; Roux et al., 2009; Uldry et al., 2012).

### “Recurrent Patterns” in AF

AF is a complex rhythm and often shows complex propagation patterns. This poses significant challenges to narrow down the potential driver sites using atrial signals often captured: (1) during single cycles of activation or short signal lengths (i.e., 2.5 s); (2) from single or localized atrial site(s); and (3) sequentially. Therefore, the search for “recurrent patterns” in AF is not a new concept but unveiling the underlying driving mechanisms from their recurrent behaviors is only possible with longer recordings from multiple atrial sites. Ng et al. proposed using “recurrence plots” of selected activations from individual EGMs in the time domain, showing checkerboard patterns of alternating high and low cross-correlation values indicating periodic recurrent patterns in the morphologies of atrial activations (Ng et al., 2014). Their approach was based on the morphologies of the EGMs which are useful for identifying recurring local activations (waves propagated from similar directions) but may not guarantee a recurrent graphic pattern with larger atrial regions involved. Simultaneous multipolar recordings were used in more recent studies, including phase mapping (Umapathy et al., 2010; Narayan et al., 2012, 2014; Rodrigo et al., 2017). Multi-site phase mapping was used to map the wave propagations, with complex patterns observed. However, stable “rotors” or phase singularities were rarely seen (Salinet et al., 2017; Podziemski et al., 2018; Li et al., 2020a) and, therefore, statistical approaches were usually employed, such as the repetitive activation patterns (RAPs) (Daoud et al., 2017), and PS density (histogram) maps (Li et al., 2020a), both able to quantify the recurrent behaviors of the core of the “rotor” visiting the same atrial location over time.

It is still debatable whether these localized rotational behaviors are true representatives of AF drivers or caused by conduction delay or blocks (Podziemski et al., 2018; Li et al., 2020b). A recently reported approach to explore recurrent patterns was the use of the CARTOFINDER system (Biosense Webster, USA) (Honarbakhsh et al., 2018). It analyses 30 s of unipolar signals obtained from the 64 poles of the basket catheters or the regional 20-pole PENTARAY catheter (Honarbakhsh et al., 2018). Recurrent rotational and focal activities can be identified from multi-site simultaneous contact EGMs. However, like other activation-based methods, the accuracy of this method is highly dependent on the robustness and accuracy of the annotation algorithm used to analyse intracardiac signals, which could be a common challenge for most time domain analyses (Tomassoni, 2017). Nevertheless, these are important studies and will be of great interest for testing the common hypothesis that atrial sites with rapid activation of highly repetitive patterns may be critical to sustaining AF. NCM has become less favored for mapping AF due to the low “morphology accuracy” for sites with electrode-to-surface distance >4 cm (Schilling et al., 1998; Thiagalingam et al., 2004; Earley et al., 2006; Shi et al., 2020). However, NCM could potentially provide ideal data for recurrent activity analysis, especially in the frequency domain (Gojraty et al., 2009). Using NCM data, the proposed method has demonstrated the ability to find recurrent spatial patterns showing interesting multi-level organization and recurrent behaviors with great potential to unveil true driving mechanisms, with the advantages of using long signal duration from a whole-chamber coverage, and without the limitation of requiring time-domain annotations.

## Limitations

This study involves a relatively small number of patients to explore the recurrent HDF patterns using high-density NCM during persAF. Nevertheless, a large number of EGMs with long signal lengths were investigated. The method proposed in the paper, like others, is reliant on predefined parameters. Further investigation on optimizing the parameters should be carried out in future work. The recurrent HDF regions found by the algorithms demonstrated organized behaviors and might be good candidates for ablation, however, this should be throughout validated and confirmed in a clinical trial.

## Conclusions

In this study, we have developed and introduced a new tool to investigate the spatiotemporal behavior of HDF in patients with persAF. Our results suggest that multiple recurrent spatiotemporal HDF patterns co-exist during persAF, with different frequencies and levels of spectral organization. The high recurrences of DP suggest a more organized rhythm. The pattern extraction algorithm can summarize the underlying non-random periodic atrial frequency activity, identifying and quantifying the spatiotemporal recurrence of the HDFs. We also found that electrograms with high organization and relative high frequency recur in time, producing similar graphic patterns. The investigation of recurrent HDF regions at different levels offer a more comprehensive dynamic insight of persAF behavior, and such regions might be good candidates for ablation.

## Data Availability Statement

The original contributions presented in the study are included in the article/Supplementary Material, further inquiries can be directed to the corresponding author.

## Ethics Statement

The studies involving human participants were reviewed and approved by the local ethics committee at the University Hospitals of Leicester NHS Trust and local NHS research ethics committee. The patients/participants provided their written informed consent to participate in this study.

## Author Contributions

XL and GC: concept/design study, data analysis/interpretation of results, drafting manuscript, critical revision of manuscript, statistics, and “off-line” data collection. TA: data analysis/interpretation of results, drafting manuscript, critical revision of manuscript, and statistics. FV, ND, ZV, and BS: data analysis/interpretation of results, critical revision of manuscript, and statistics. JS and AM: data analysis/interpretation of results and critical revision of manuscript. PS: EP study, data collection, interpretation of results, and critical revision of manuscript. FS: concept/design study, data analysis/interpretation of results, and critical revision of manuscript. GN: EP studies and ablation procedures, concept/design study, interpretation of results, and critical revision of manuscript. All authors: contributed to the article and approved the submitted version.

## Conflict of Interest

GN has received a research fellowship from St. Jude Medical (now Abbott) and speaker fees and honoraria from Biosense Webster. The remaining authors declare that the research was conducted in the absence of any commercial or financial relationships that could be construed as a potential conflict of interest. The handling Editor declared a past co-authorship with one of the authors TA.
