# Supplementary material for: Automatic Extraction of Recurrent Patterns of High Dominant Frequency Mapping During Human Persistent Atrial Fibrillation
Source: Front Physiol. 2021 Mar 12;12:649486. doi: 10.3389/fphys.2021.649486 (PMC7994862; doi:10.3389/fphys.2021.649486)
Supplement: Supplementary file 1 [file Table_1.DOCX]

Supplementary Material

Automatic Extraction of Recurrent Patterns of High Dominant Frequency Mapping during Human Persistent Atrial Fibrillation

Xin Li*^1,2^, Gavin S Chu*^1^, Tiago P Almeida^1,2^, Frederique J Vanheusden^3^, João Salinet^4^, Nawshin Dastagir^5^, Amar R Mistry^1^ , Zakariyya Vali^1^, Bharat Sidhu^1^, Peter J Stafford^6^, Fernando S Schlindwein^2,6^, G André Ng^1,6^

^1^Department of Cardiovascular Science, University of Leicester, UK;

^2^School of Engineering, University of Leicester, UK;

^3^School of Science & Technology, Nottingham Trent University, UK;

^4^Biomedical Engineering, Centre for Engineering, Modelling and Applied Social Sciences (CECS), Federal University of ABC, Brazil; ^5^Auckland Bioengineering Institute, University of Auckland, New Zealand

^6^National Institute for Health Research Leicester Cardiovascular Biomedical Research Centre, Glenfield Hospital, UK

*** Correspondence:**

Dr. Xin Li,

Department of Cardiovascular Sciences/School of Engineering

University of Leicester, LE1 7RH, UK

Tel: +44 (0) 116 229 7380

Email: [xl251@leicester.ac.uk](mailto:xl251@leicester.ac.uk)

* These authors contributed equally to the manuscript

Keywords: Atrial fibrillation, catheter ablation, non-contact mapping, atrial electrograms, dominant frequency, recurrent patterns, spatiotemporal patterns

# Supplementary Data

**
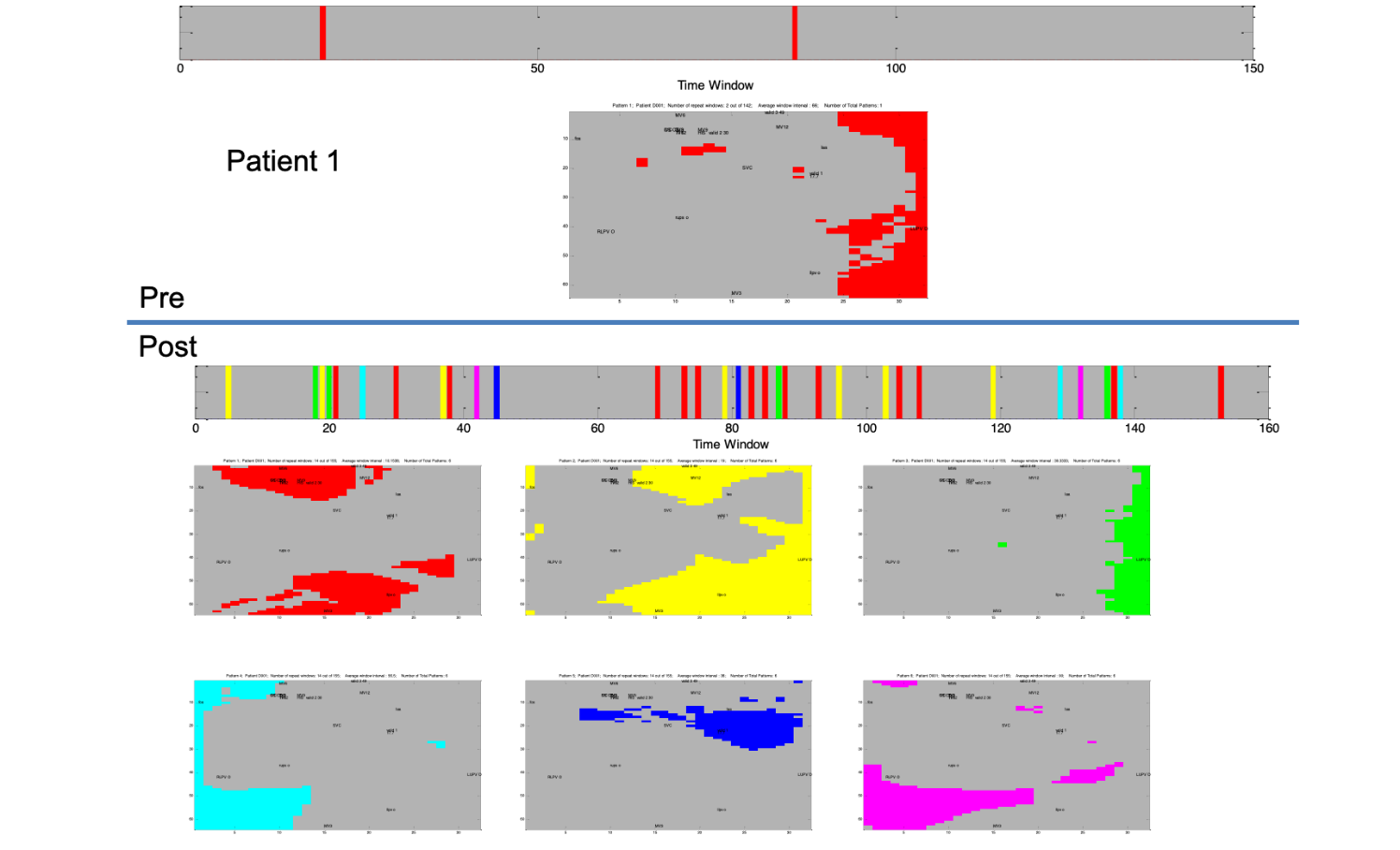
**

Figure S1 The all dominant and secondary dominant patterns of patient 1 in 2D and their corresponding occurrences in time colour-coded by different patterns pre-/post- ablation

**
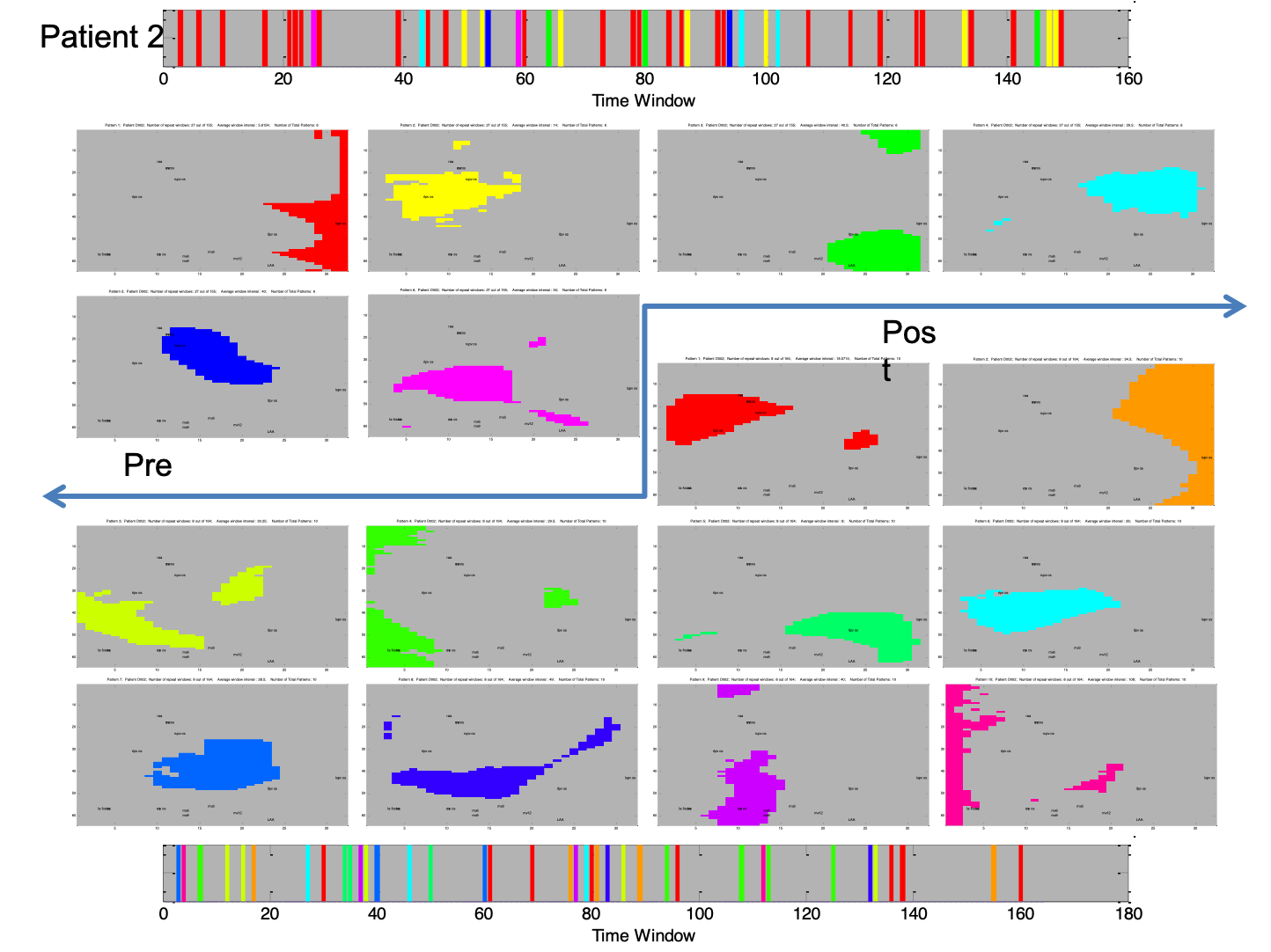
**

Figure S2 The all dominant and secondary dominant patterns of patient 2 in 2D and their corresponding occurrences in time colour-coded by different patterns pre-/post- ablation

**
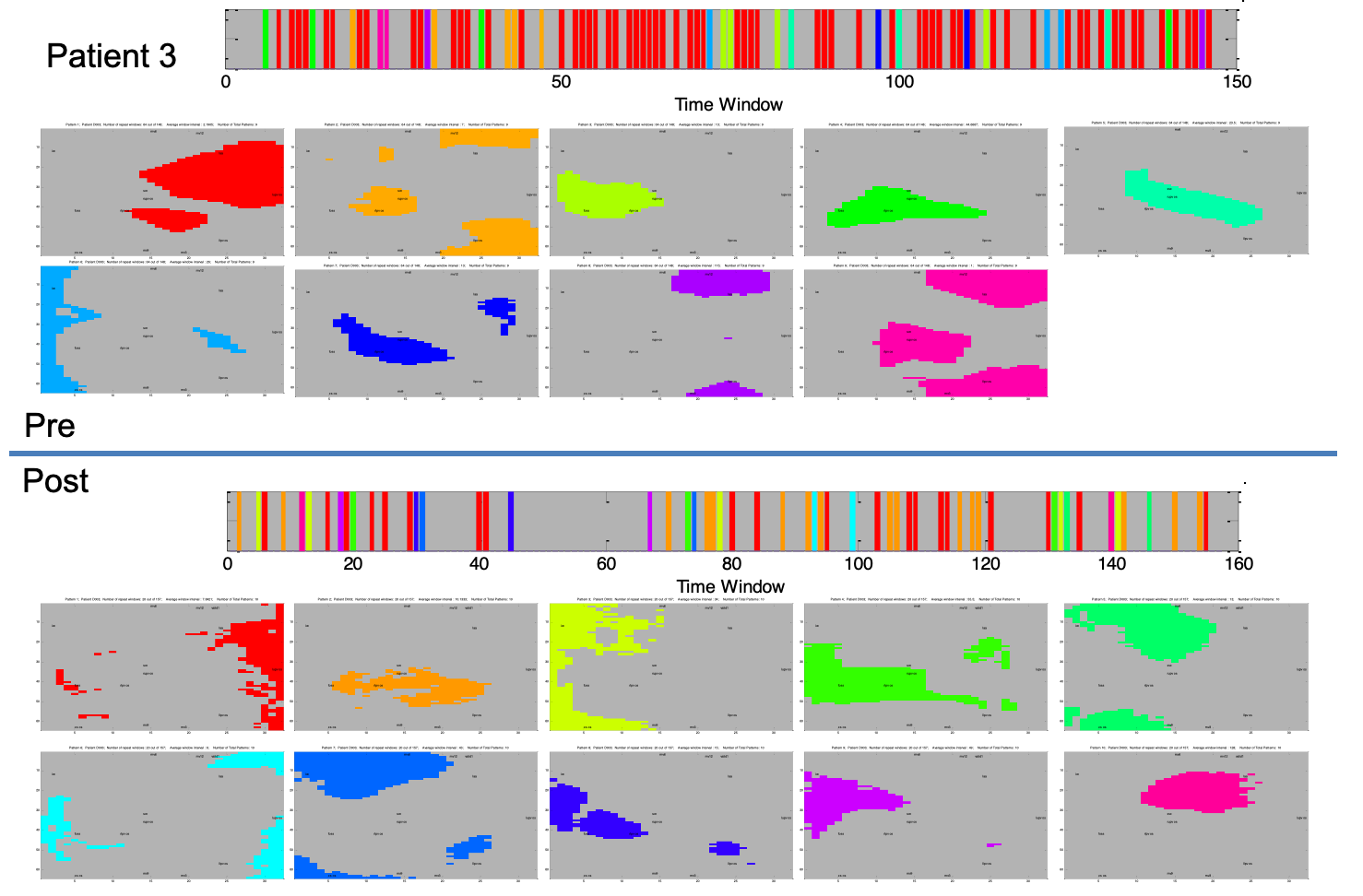
**

Figure S3 The all dominant and secondary dominant patterns of patient 3 in 2D and their corresponding occurrences in time colour-coded by different patterns pre-/post- ablation

**
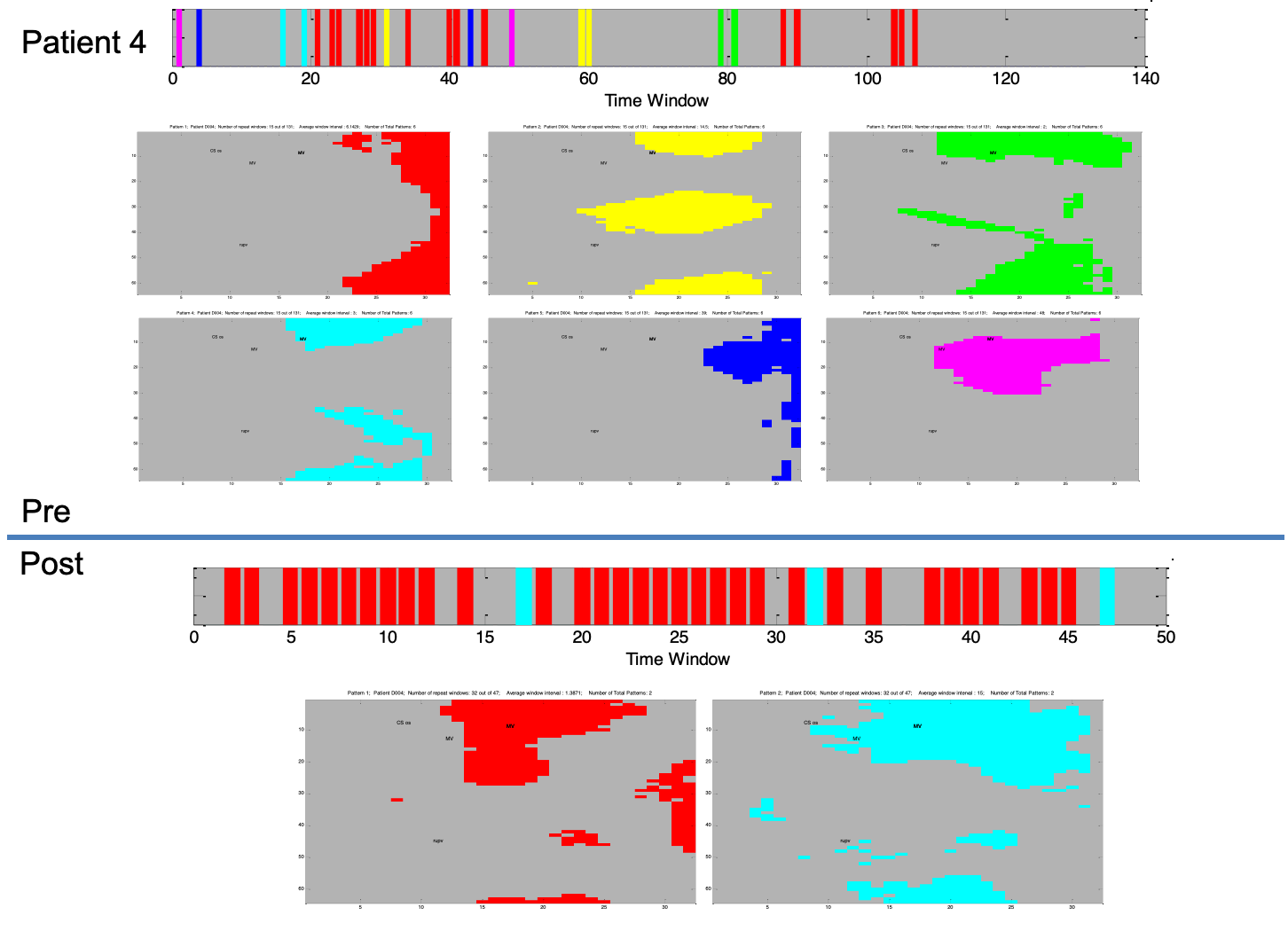
**

Figure S4 The all dominant and secondary dominant patterns of patient 4 in 2D and their corresponding occurrences in time colour-coded by different patterns pre-/post- ablation

**
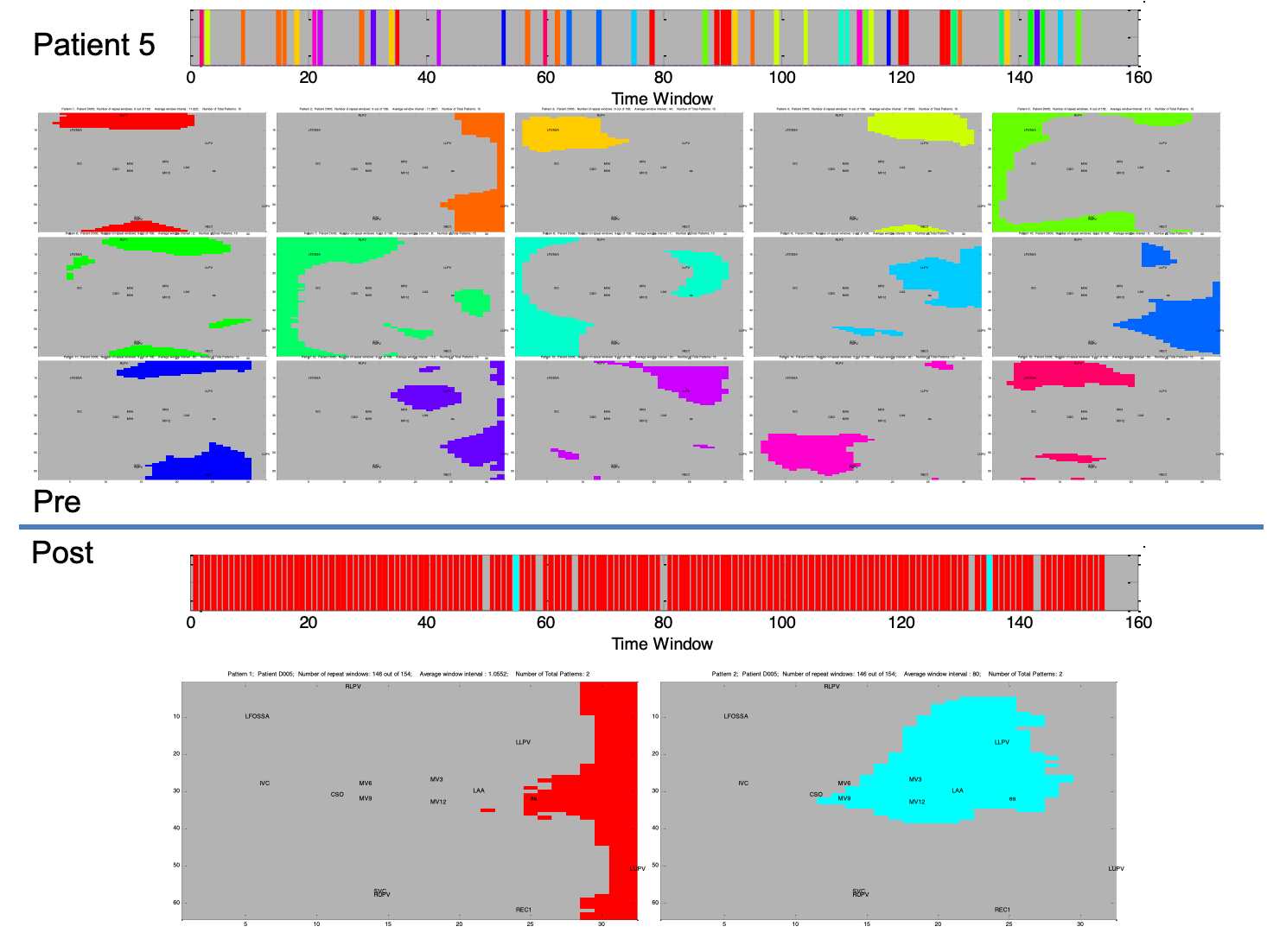
**

Figure S5 The all dominant and secondary dominant patterns of patient 5 in 2D and their corresponding occurrences in time colour-coded by different patterns pre-/post- ablation

**
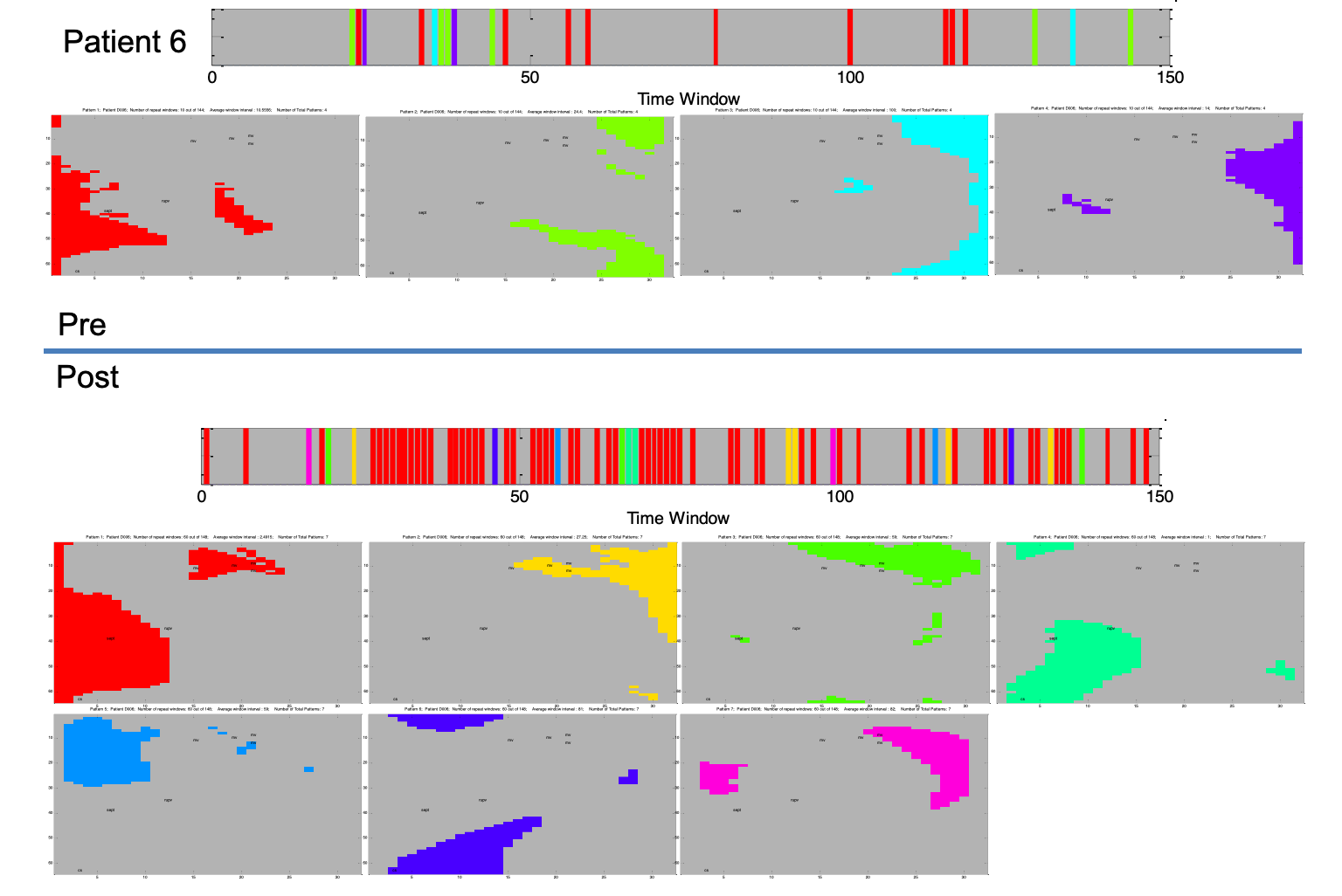
**

Figure S6 The all dominant and secondary dominant patterns of patient 6 in 2D and their corresponding occurrences in time colour-coded by different patterns pre-/post- ablation

**
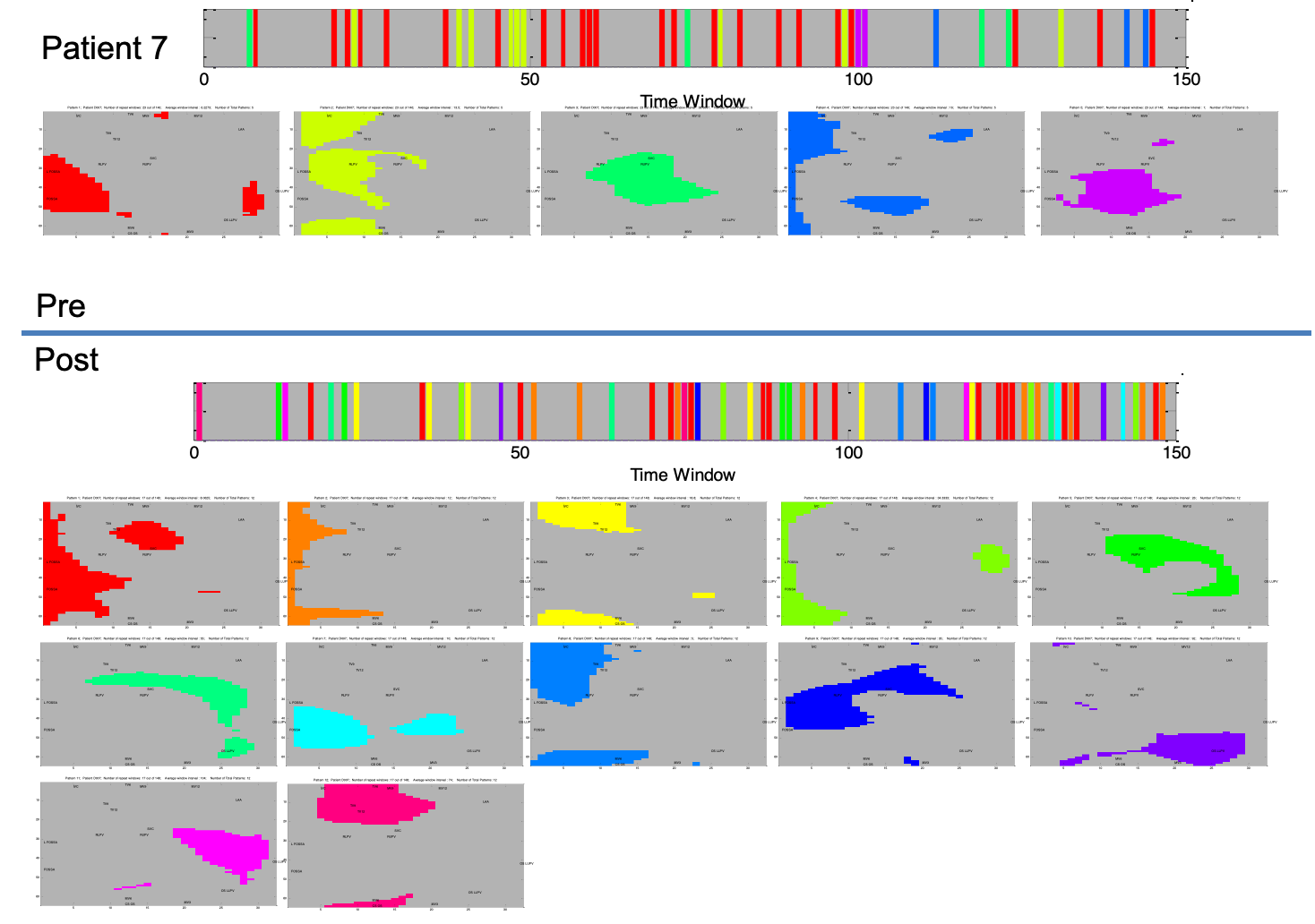
**

Figure S7 The all dominant and secondary dominant patterns of patient 7 in 2D and their corresponding occurrences in time colour-coded by different patterns pre-/post- ablation

**
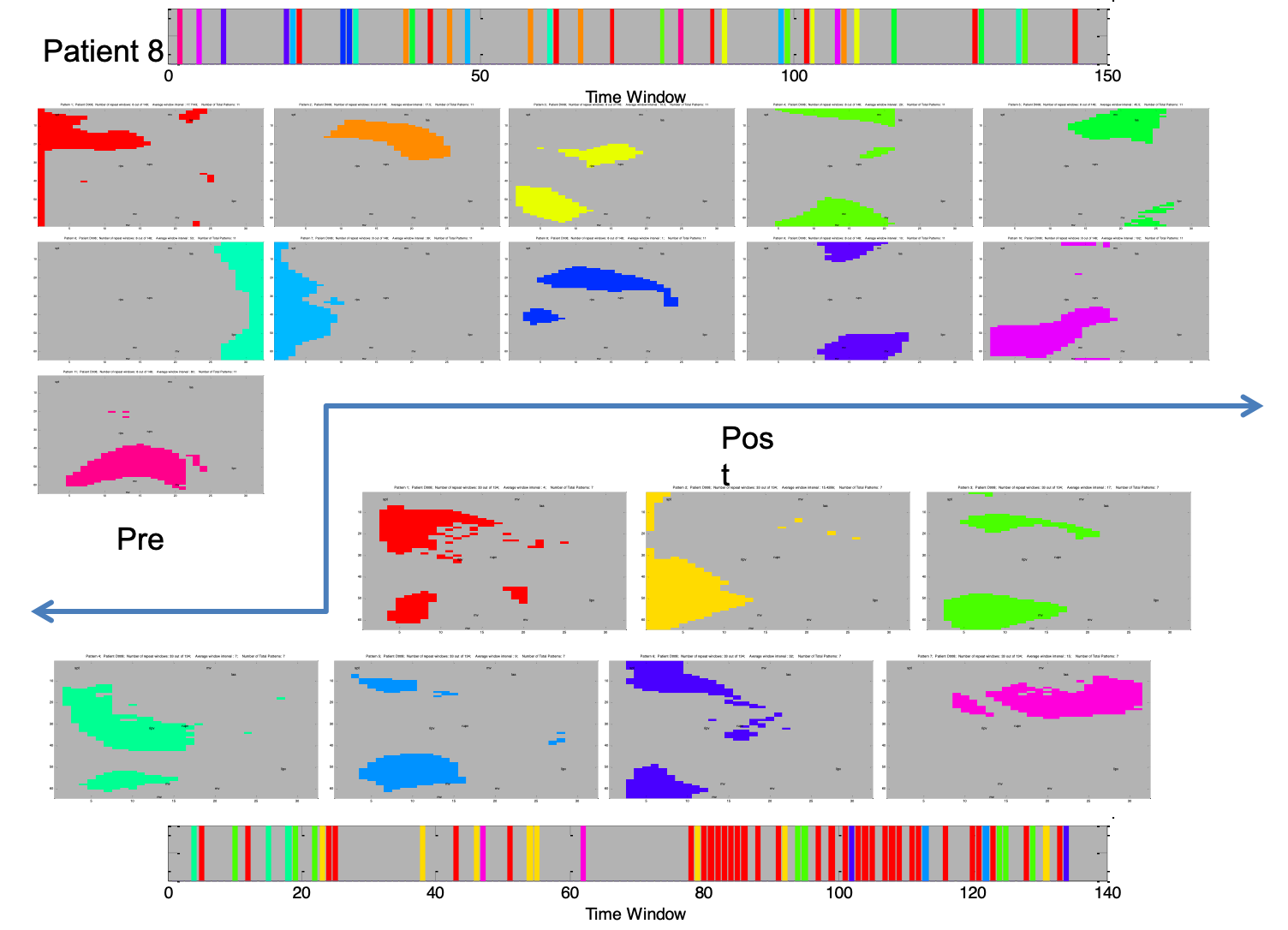
**

Figure S8 The all dominant and secondary dominant patterns of patient 8 in 2D and their corresponding occurrences in time colour-coded by different patterns pre-/post- ablation

**
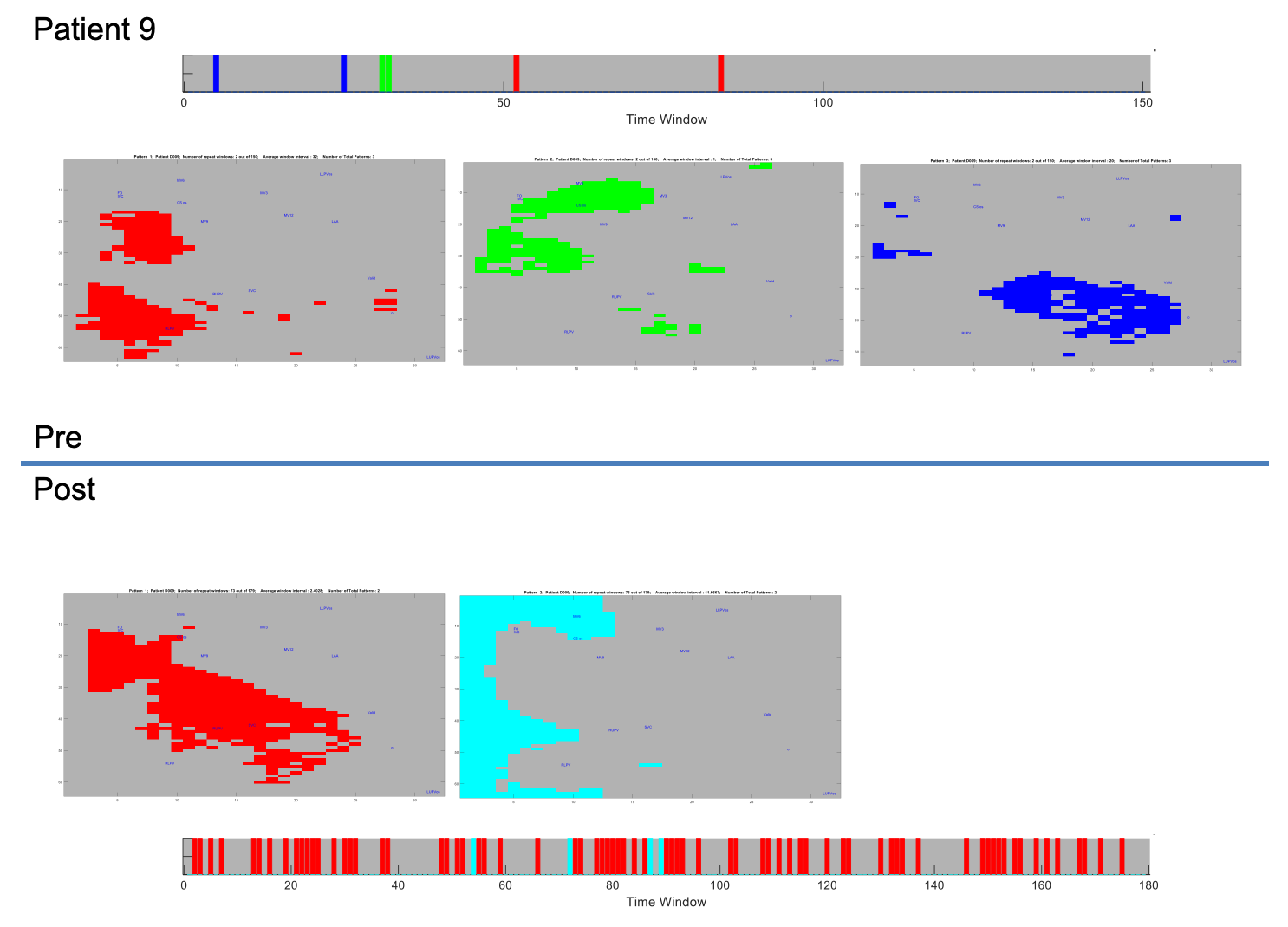
**

Figure S9 The all dominant and secondary dominant patterns of patient 9 in 2D and their corresponding occurrences in time colour-coded by different patterns pre-/post- ablation

**
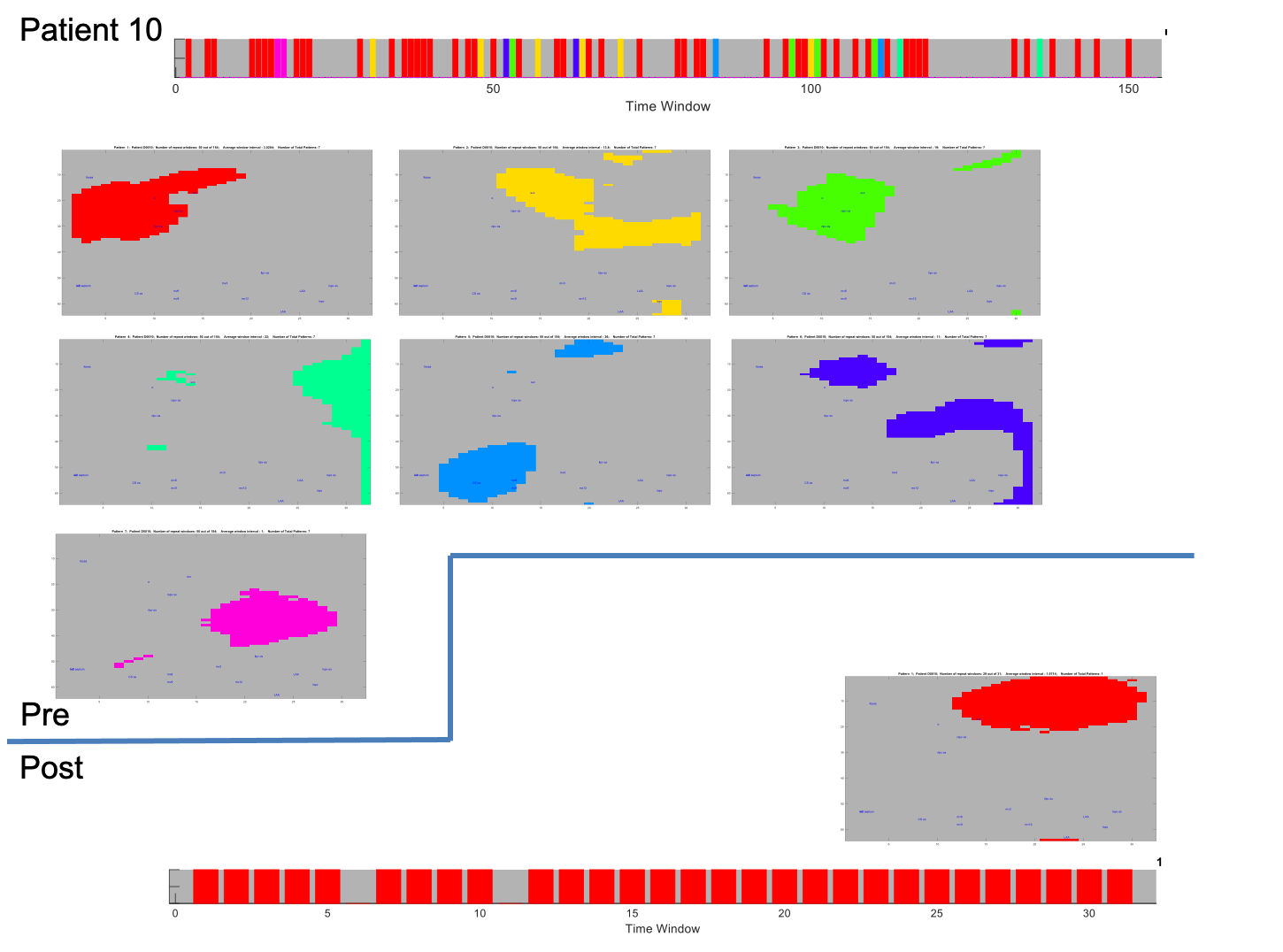
**

Figure S10 The all dominant and secondary dominant patterns of patient 10 in 2D and their corresponding occurrences in time colour-coded by different patterns pre-/post- ablation

**Effect of changing the Correlation Coefficient (CORR) Threshold**

**Figure S11** shows the relationship between the fraction of number of time windows in DP over total number of windows and the CORR threshold used in the algorithm. As expected, the number of windows reduces when the threshold of the pattern extraction algorithm increases.


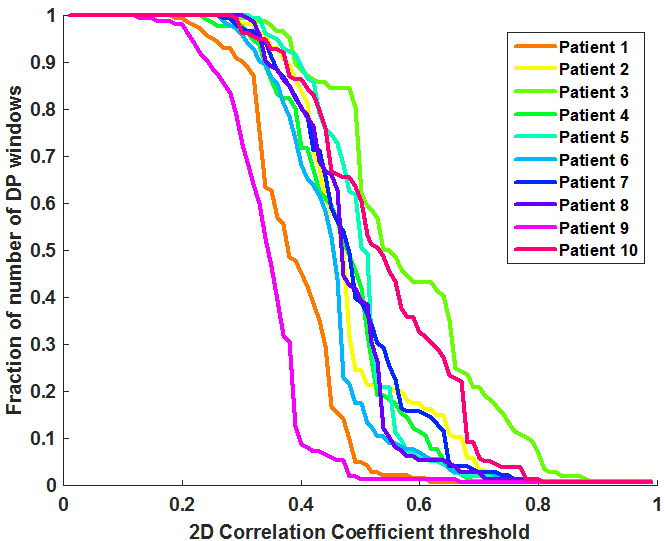


**Figure S11** the relationship between the fraction of number of time windows in DP over total number of windows and the CORR threshold used in the algorithm.

## Retrospective database

The recurrent patterns and the proposed algorithm represent a fully retrospective analysis, independent from the ablation strategy. The ablation was guided by targeting the high DF sites with an in-house MATLAB GUI described previously (1), targeting the clusters of HDF centres. As illustrated in **Figure S12 A**, the MATLAB GUI is showing front (left sub-panel) and back (right sub-panel) views of HDF trajectory map. In the left map, the dark red regions are the HDF region of the current time window and the centre of this HDF regions is marked with a white dot. However, HDF maps are not stable and can move across the atrium in different time windows. There were other HDF regions in previous time windows where their centres were marked with pink dots and the trajectory of travel of the HDF centres were marked with the smaller dotted line in white. The HDF region in each time window was defined as the atrial locations with DF values that are greater or equal to the 0.25Hz below the maximum DF across all the 2048 locations (DFmax - 0.25Hz) as previously described (1) .

Ablation targets (black circled region in **Figure S12 B**) were defined as the clusters of the HDF centres. Usually, electrophysiologist and team will mark a few ablation targets and ranked by priority after viewing the clusters of the HDF centres from the Matlab GUI. The black line regions were created by the Abbott EP engineer on the Ensite mapping system following guidance of the electrophysiologist.


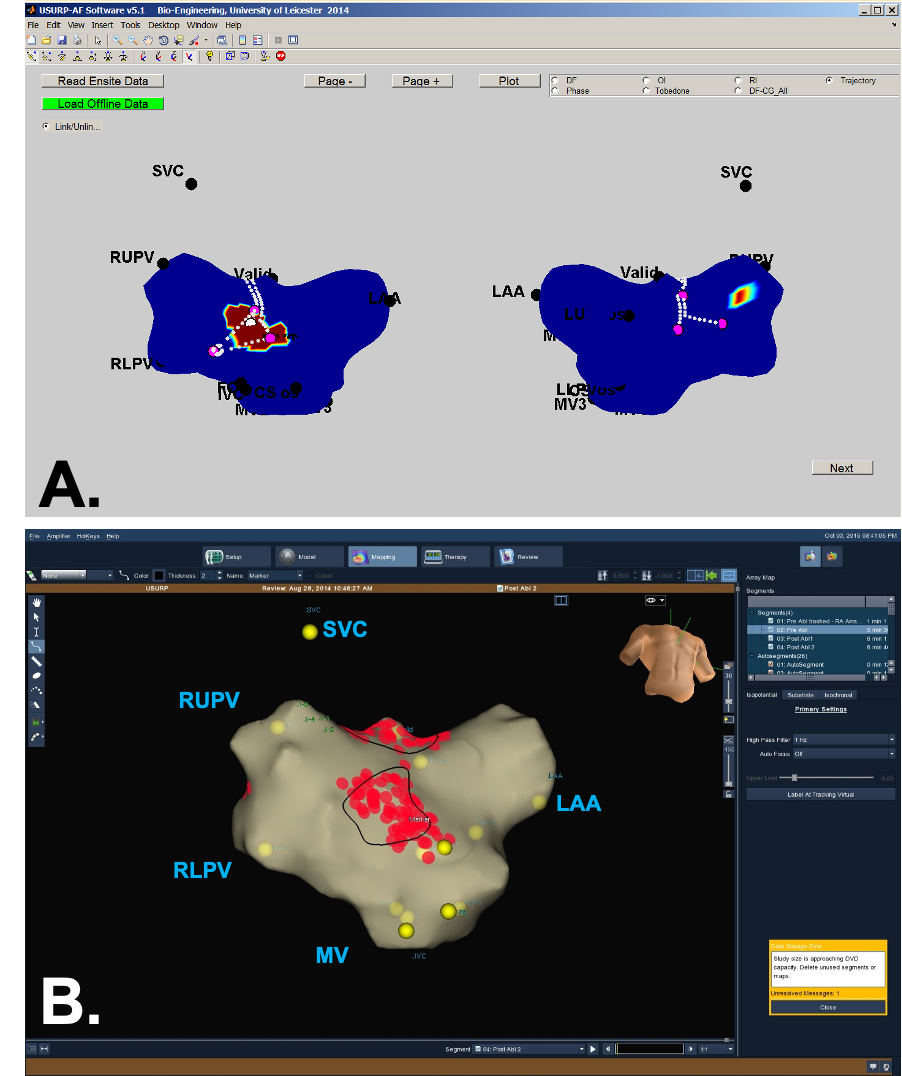


**Figure S12** Example of in-house software and ablation strategy of the database used in current work. The clusters of centres of HDF sites were targeted for ablation. **A.** Our MATLAB GUI showing front (left sub-panel) and back (right sub-panel) views of HDF trajectory map (dark red region: HDF, pink dots: previous centres of HDF areas, large white dot: current centre of HDF area, small white dotted lines: trajectory of centres of HDF areas movement over time); **B.** Ensite system map with ablation target (black line area) and ablation lesions (red dots)**.**

**Figures S13 - S22** illustrate the recurrent patterns and the ablation lesions (guided by DF) for all patients. Please note, ablation was guided by high DF sites (1), and not by recurrent HDF patterns. Further prospective study targeting recurrent pattens may be needed.

**
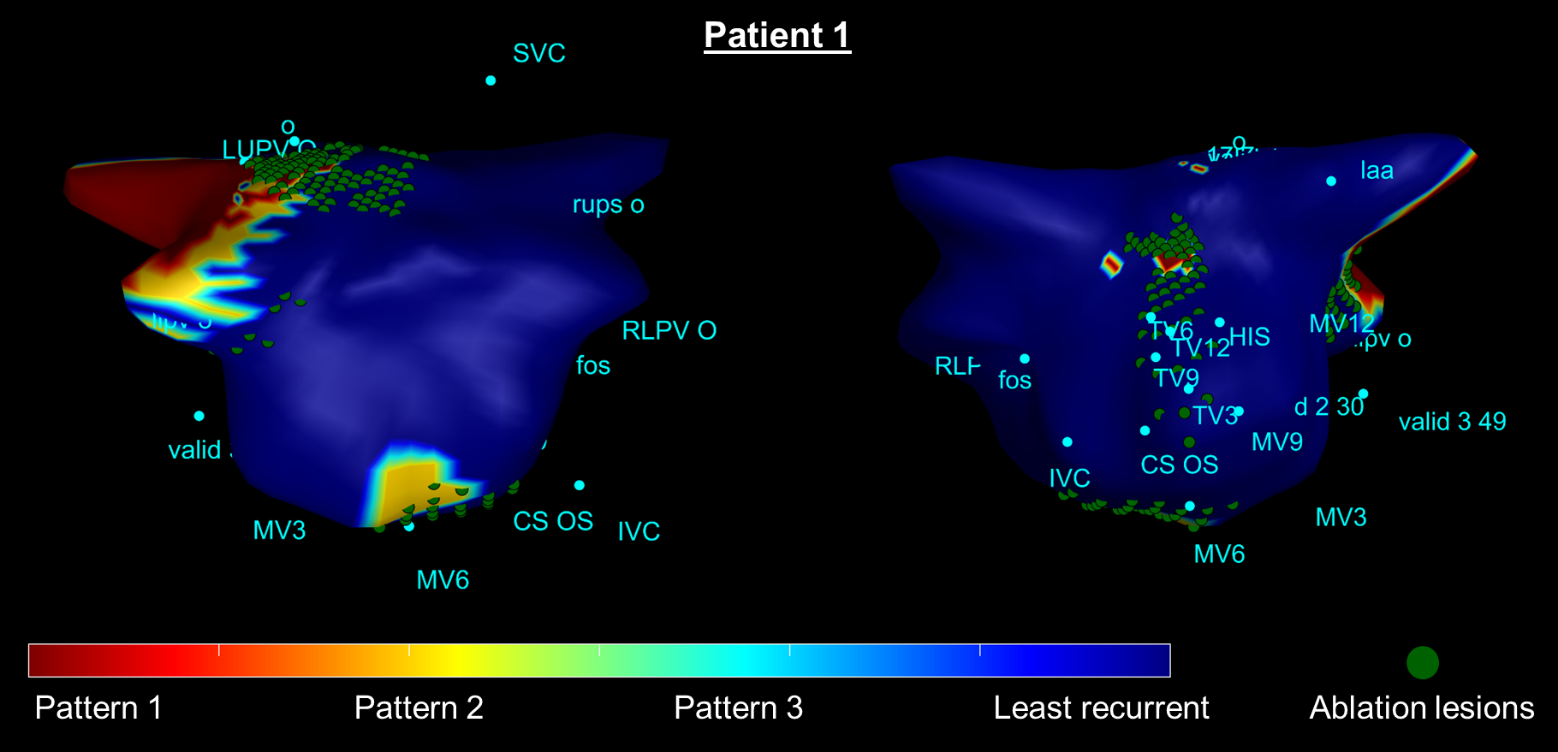
**

## Figure S13 Patient 1 - 3D LA map (two views) reconstructed in MATLAB, colour-coded with top 3 recurrent patterns and ranked by occurrences (Red: Pattern 1 (most recurrent), Yellow: Pattern 2, light blue (Pattern 3), dark blue: least recurrent region). The green dots represent the estimated ablation lesion (red dots in Figure S12 B) locations projected on the 3D geometry surface in MATLAB.


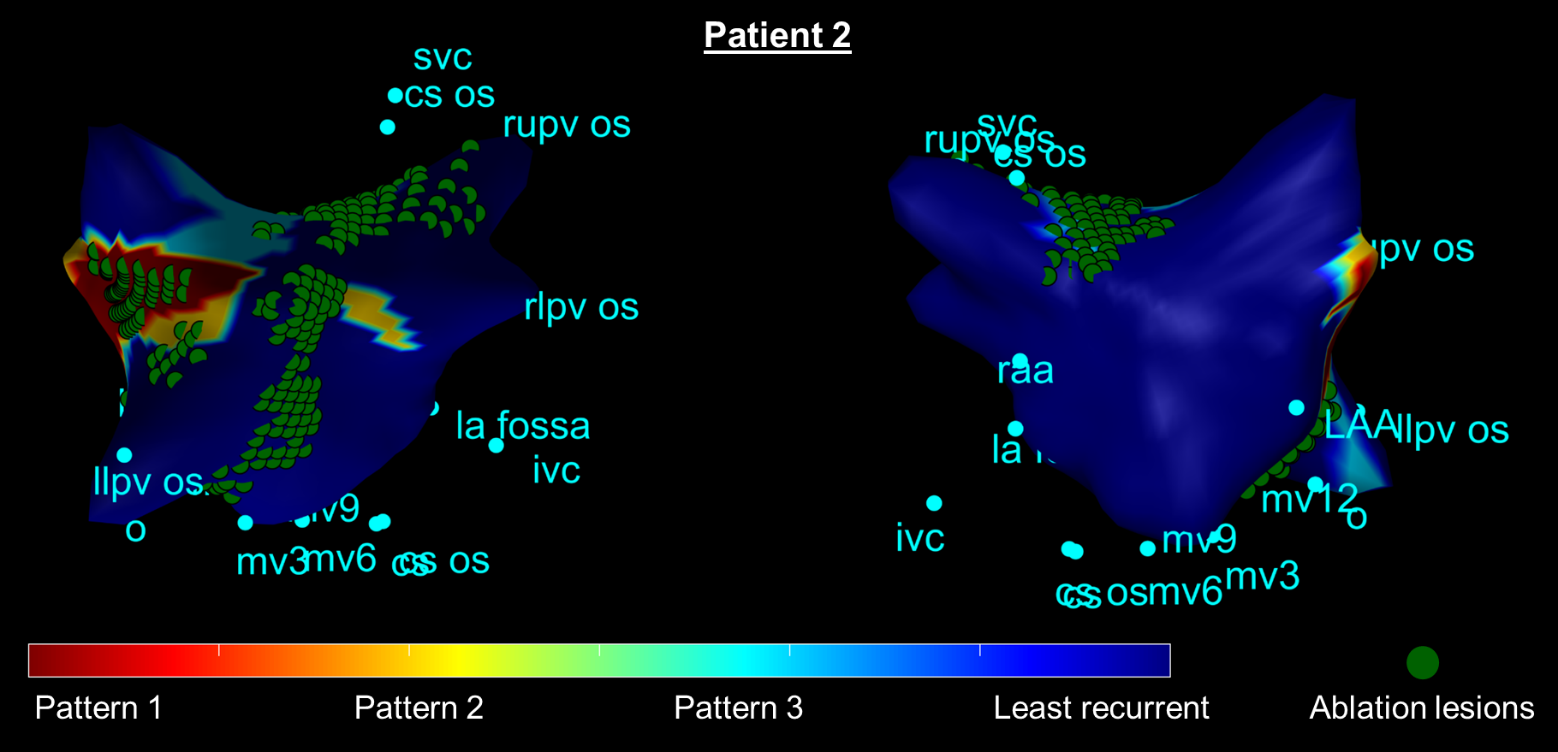


## Figure S14 Patient 2 - 3D LA map (two views) reconstructed in MATLAB, colour-coded with top 3 recurrent patterns and ranked by occurrences (Red: Pattern 1 (most recurrent), Yellow: Pattern 2, light blue (Pattern 3), dark blue: least recurrent region). The green dots represent the estimated ablation lesion (red dots in Figure S12 B) locations projected on the 3D geometry surface in MATLAB.


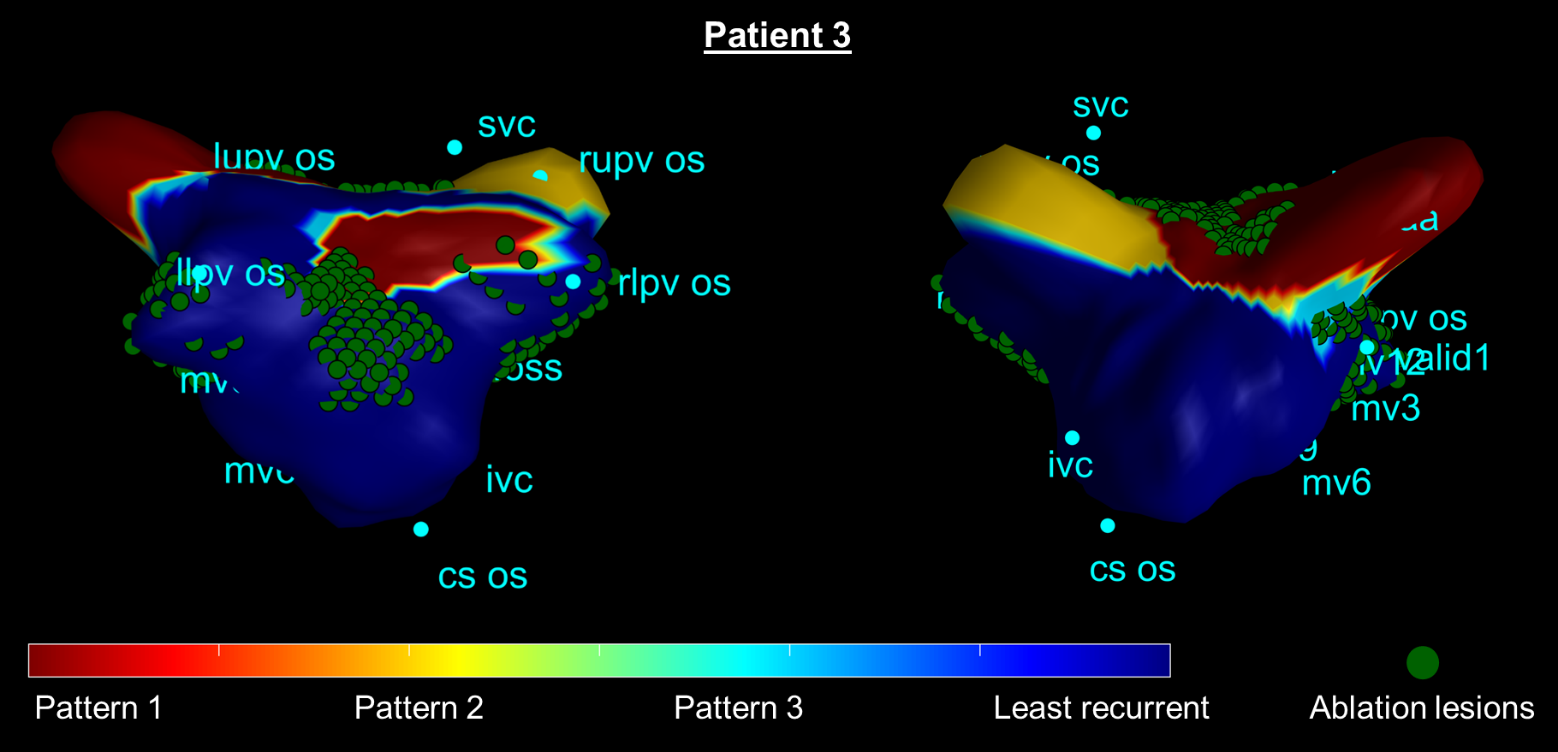


## Figure S15 Patient 3 - 3D LA map (two views) reconstructed in MATLAB, colour-coded with top 3 recurrent patterns and ranked by occurrences (Red: Pattern 1 (most recurrent), Yellow: Pattern 2, light blue (Pattern 3), dark blue: least recurrent region). The green dots represent the estimated ablation lesion (red dots in Figure S12 B) locations projected on the 3D geometry surface in MATLAB.


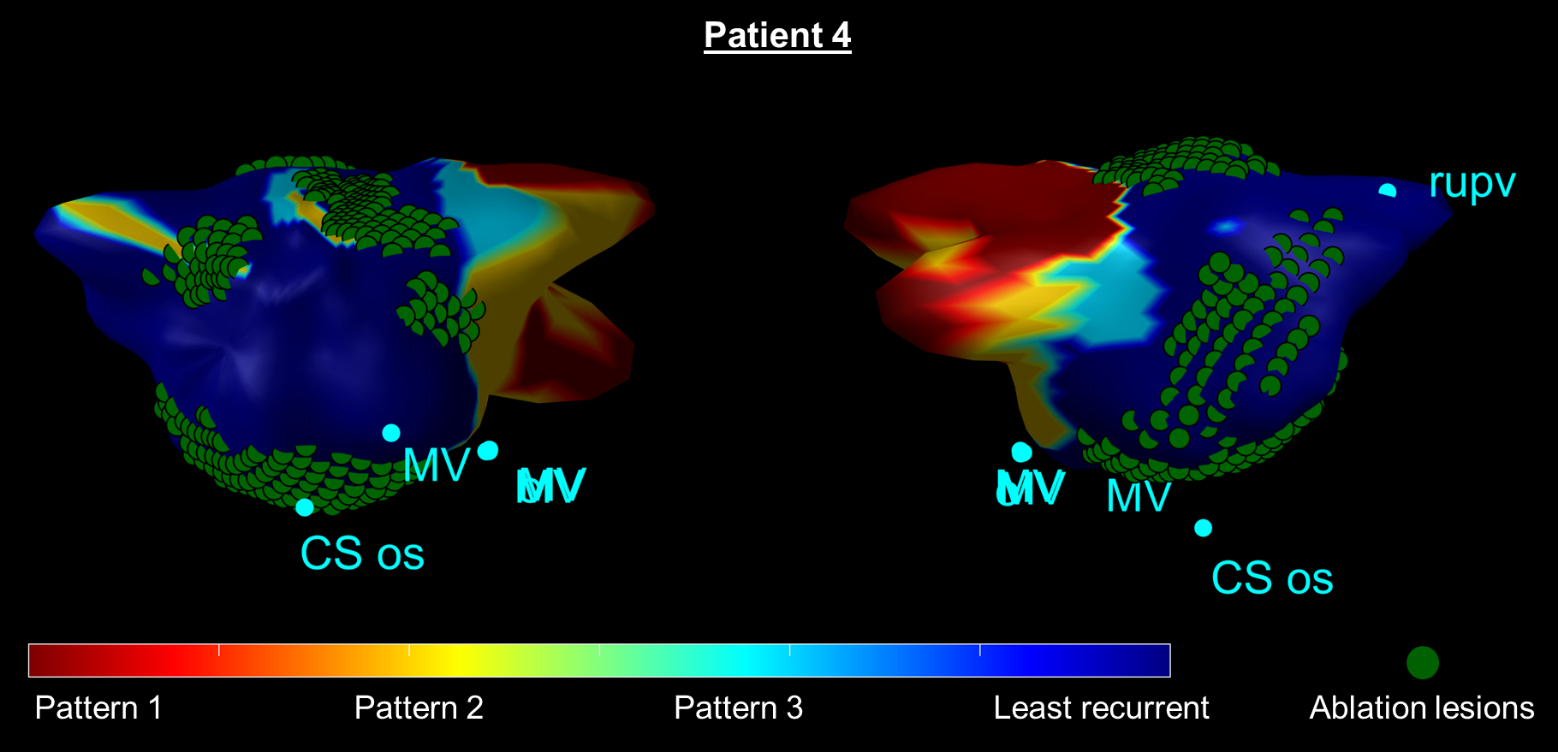


## Figure S16 Patient 4 - 3D LA map (two views) reconstructed in MATLAB, colour-coded with top 3 recurrent patterns and ranked by occurrences (Red: Pattern 1 (most recurrent), Yellow: Pattern 2, light blue (Pattern 3), dark blue: least recurrent region). The green dots represent the estimated ablation lesion (red dots in Figure S12 B) locations projected on the 3D geometry surface in MATLAB.


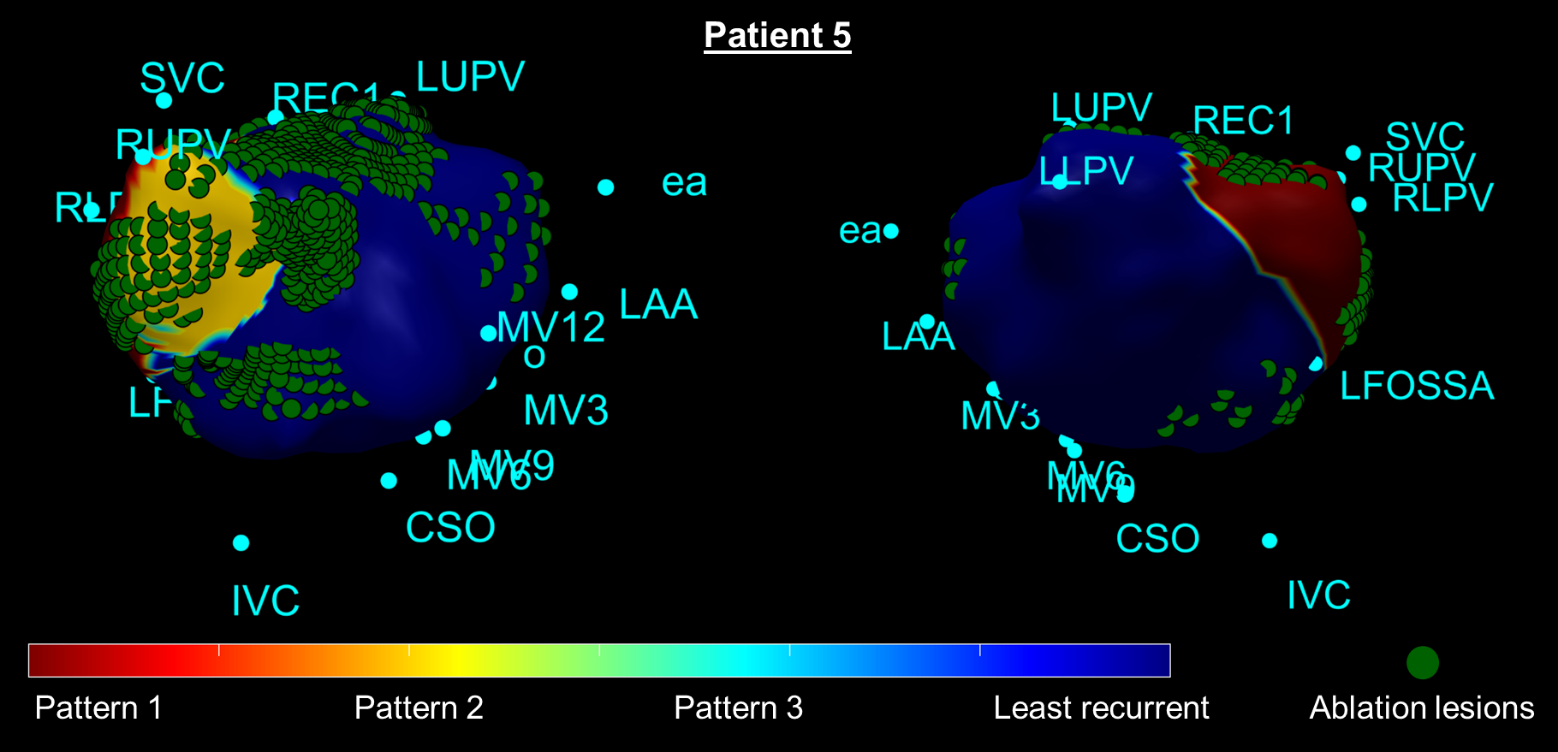


## Figure S17 Patient 5 - 3D LA map (two views) reconstructed in MATLAB, colour-coded with top 3 recurrent patterns and ranked by occurrences (Red: Pattern 1 (most recurrent), Yellow: Pattern 2, light blue (Pattern 3), dark blue: least recurrent region). The green dots represent the estimated ablation lesion (red dots in Figure S12 B) locations projected on the 3D geometry surface in MATLAB.


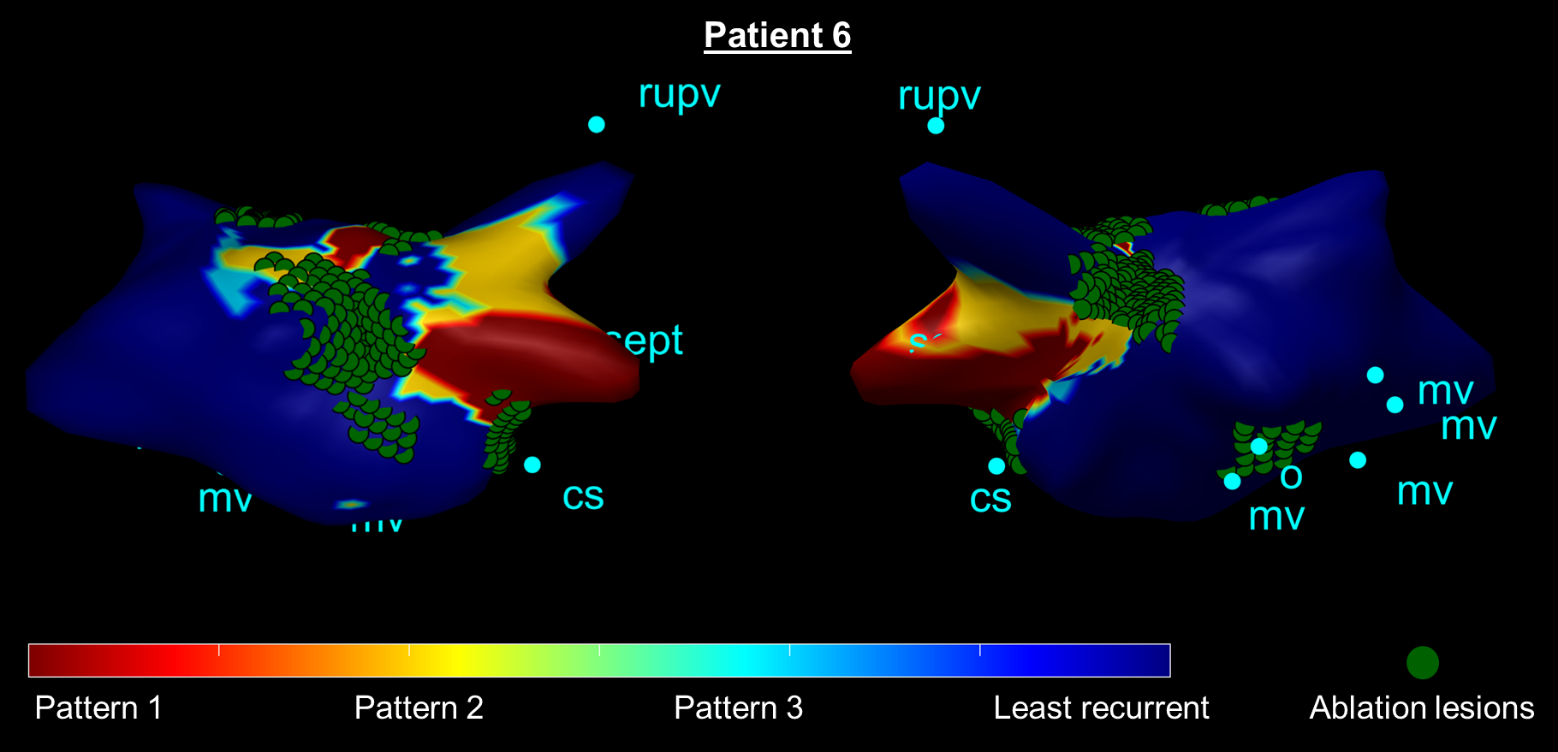


## Figure S18 Patient 6 - 3D LA map (two views) reconstructed in MATLAB, colour-coded with top 3 recurrent patterns and ranked by occurrences (Red: Pattern 1 (most recurrent), Yellow: Pattern 2, light blue (Pattern 3), dark blue: least recurrent region). The green dots represent the estimated ablation lesion (red dots in Figure S12 B) locations projected on the 3D geometry surface in MATLAB.


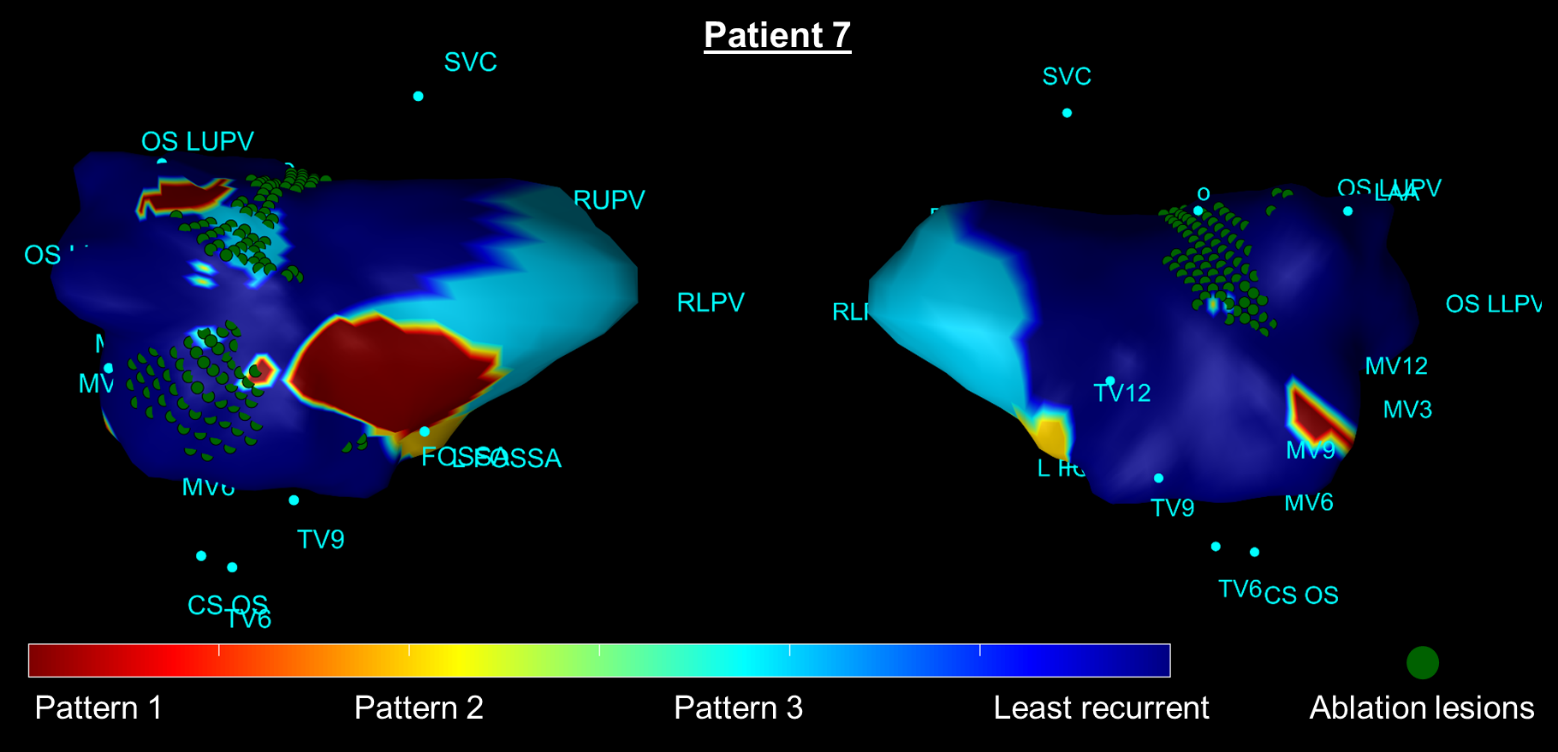


## Figure S19 Patient 7 - 3D LA map (two views) reconstructed in MATLAB, colour-coded with top 3 recurrent patterns and ranked by occurrences (Red: Pattern 1 (most recurrent), Yellow: Pattern 2, light blue (Pattern 3), dark blue: least recurrent region). The green dots represent the estimated ablation lesion (red dots in Figure S12 B) locations projected on the 3D geometry surface in MATLAB.


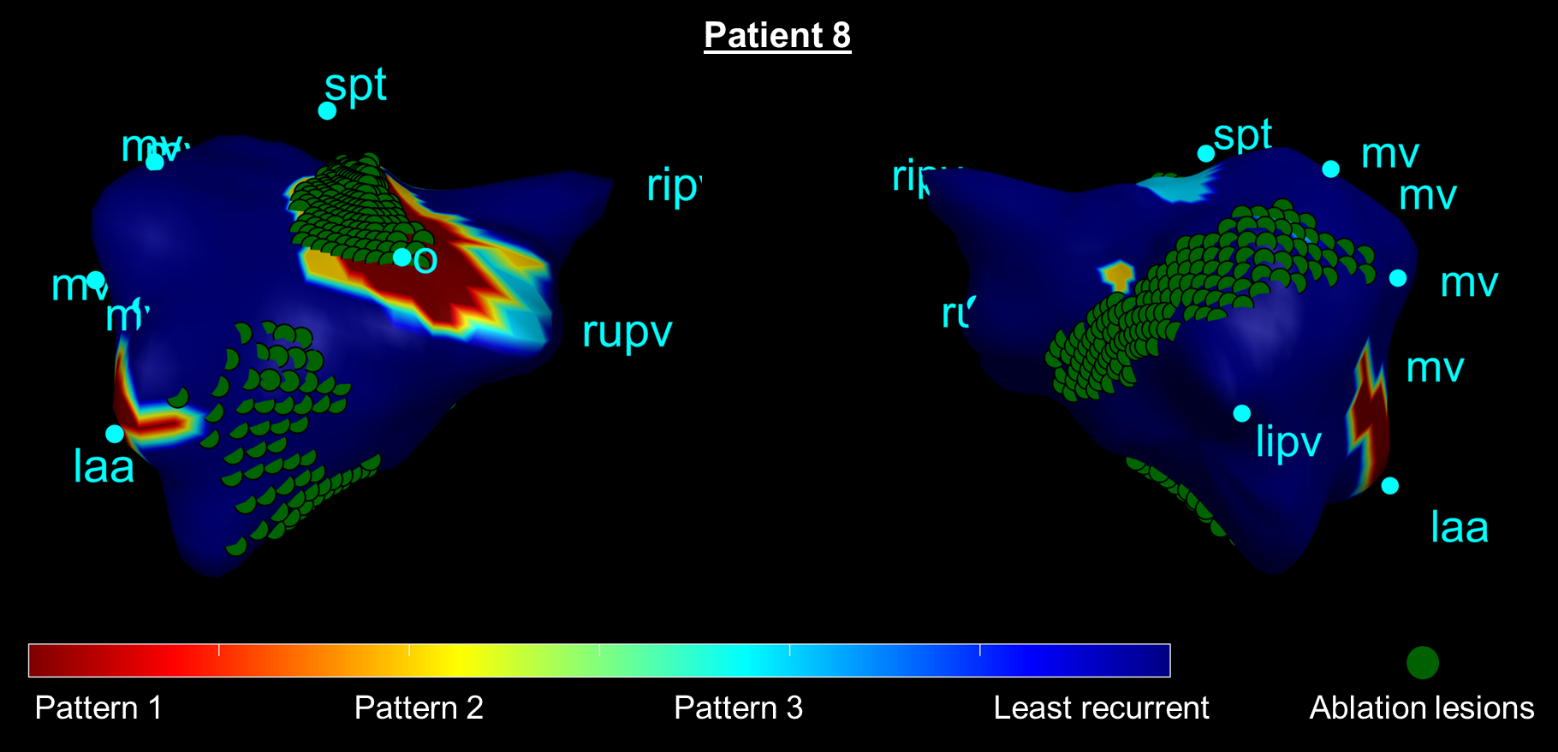


## Figure S20 Patient 8 - 3D LA map (two views) reconstructed in MATLAB, colour-coded with top 3 recurrent patterns and ranked by occurrences (Red: Pattern 1 (most recurrent), Yellow: Pattern 2, light blue (Pattern 3), dark blue: least recurrent region). The green dots represent the estimated ablation lesion (red dots in Figure S12 B) locations projected on the 3D geometry surface in MATLAB.


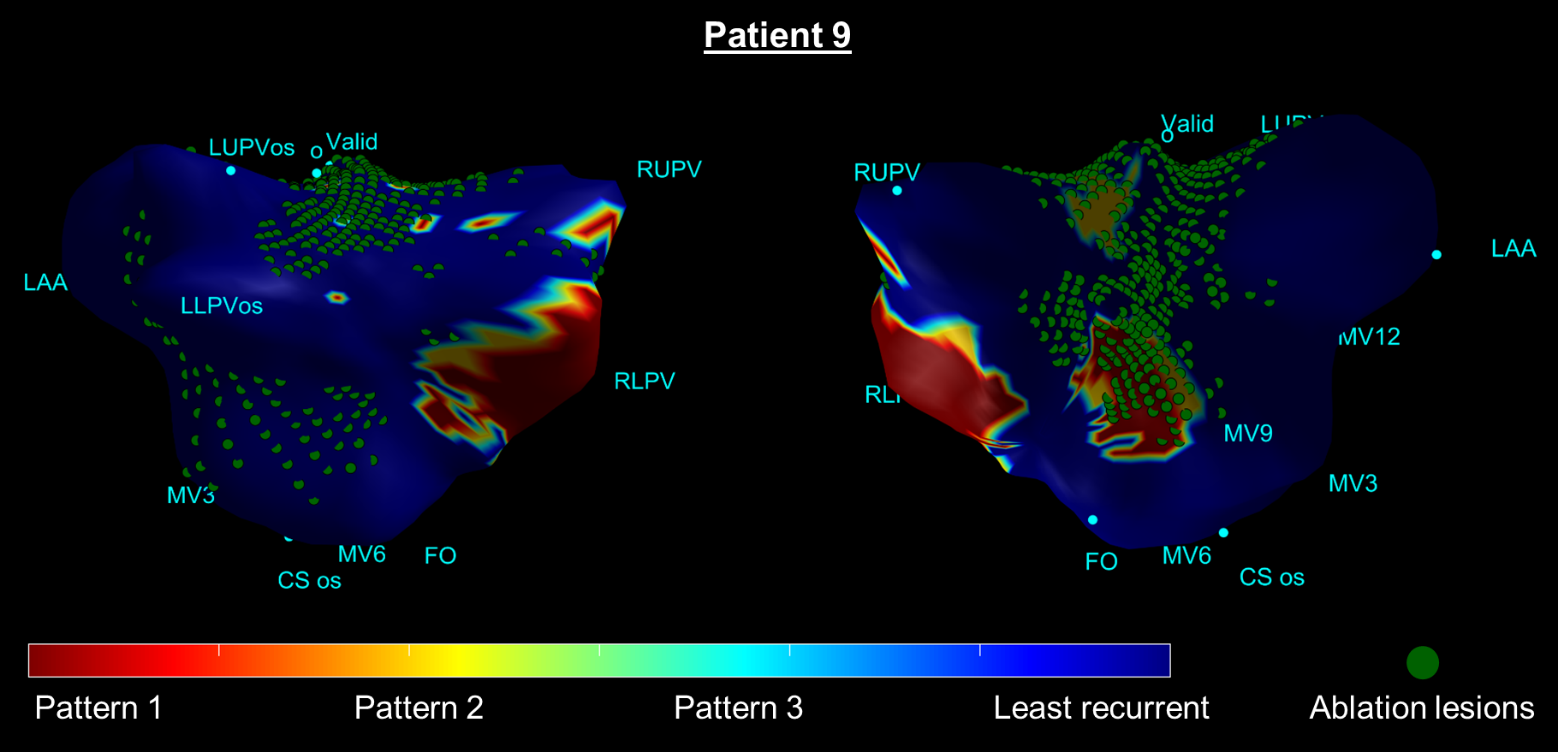


## Figure S21 Patient 9 - 3D LA map (two views) reconstructed in MATLAB, colour-coded with top 3 recurrent patterns and ranked by occurrences (Red: Pattern 1 (most recurrent), Yellow: Pattern 2, light blue (Pattern 3), dark blue: least recurrent region). The green dots represent the estimated ablation lesion (red dots in Figure S12 B) locations projected on the 3D geometry surface in MATLAB.


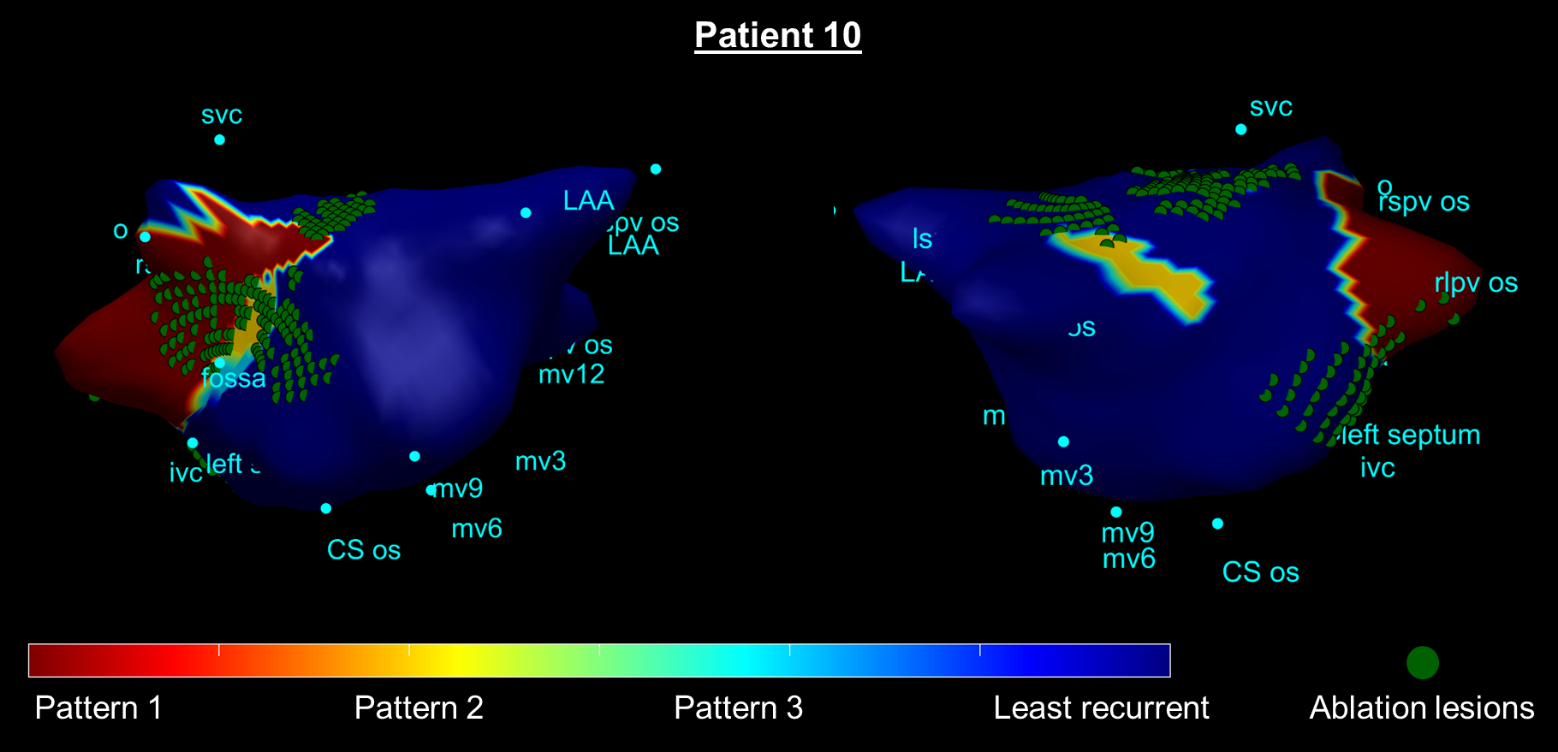


## Figure S22 Patient 10 - 3D LA map (two views) reconstructed in MATLAB, colour-coded with top 3 recurrent patterns and ranked by occurrences (Red: Pattern 1 (most recurrent), Yellow: Pattern 2, light blue (Pattern 3), dark blue: least recurrent region). The green dots represent the estimated ablation lesion (red dots in Figure S12 B) locations projected on the 3D geometry surface in MATLAB.

## DF mapping using NCM

Previous studies showed moderate correlation between non-contact and contact mapping (2-4). Schilling and colleagues found a correlation of 0.74±0.19 for 3600 EGMs tested in the right atrium (2); Earley *et al.* showed similar correlation of 0.81 (0.27 to 0.98) from the LA (3); Jarman and colleagues showed a correlation of 0.70±0.15 for 62 random locations in the LA (4); finally, it was also shown that correlation decreased with increasing distance between the endocardial node and the balloon (3, 5, 6). More recently, a more advanced noncontact dipole density mapping system was validated and generated a comparable result (7). These comparisons, however, were limited on the correlation of the EGMs’ morphology. The use of NCM in the frequency domain was validated by Gojraty *et al.*, where no significant difference was found in the mean DFs between contact and noncontact signals (8). Higher correlation in frequency domain (8) may still suggest non-contact mapping to be a good mapping tool with the advantages of whole-chamber coverage when mapping the recurrent frequency patterns.

**References**

1. Li X, Salinet JL, Almeida TP, Vanheusden FJ, Chu GS, Ng GA, et al. An interactive platform to guide catheter ablation in human persistent atrial fibrillation using dominant frequency, organization and phase mapping. Comput Methods Programs Biomed. 2017;141:83-92.

2. Schilling RJ, Kadish AH, Peters NS, Goldberger J, Davies DW. Endocardial mapping of atrial fibrillation in the human right atrium using a non-contact catheter. European Heart Journal. 2000;21(7):550-64.

3. Earley MJ, Abrams DJ, Sporton SC, Schilling RJ. Validation of the noncontact mapping system in the left atrium during permanent atrial fibrillation and sinus rhythm. J Am Coll Cardiol. 2006;48(3):485-91.

4. Jarman JWE, Wong T, Kojodjojo P, Spohr H, Davies JE, Roughton M, et al. Spatiotemporal behavior of high dominant frequency during paroxysmal and persistent atrial fibrillation in the human left atrium. Circulation: Arrhythmia and Electrophysiology. 2012;5(4):650-8.

5. Schilling RJ, Peters NS, Davies W. Simultaneous endocardial mapping in the human left ventricle using a noncontact catheter - Comparison of contact and reconstructed electrograms during sinus rhythm. Circulation. 1998;98(9):887-98.

6. Thiagalingam A, Wallace EM, Boyd AC, Eipper VE, Campbell CR, Byth K, et al. Noncontact mapping of the left ventricle: insights from validation with transmural contact mapping. Pace. 2004;27(5):570-8.

7. Shi R, Parikh P, Chen Z, Angel N, Norman M, Hussain W, et al. Validation of Dipole Density Mapping During Atrial Fibrillation and Sinus Rhythm in Human Left Atrium. JACC Clin Electrophysiol. 2020;6(2):171-81.

8. Gojraty S, Lavi N, Valles E, Kim SJ, Michele J, Gerstenfeld EP. Dominant frequency mapping of atrial fibrillation: comparison of contact and noncontact approaches. J Cardiovasc Electrophysiol. 2009;20(9):997-1004.
